# Experimental evolution of a mammalian holobiont: bank voles selected for herbivorous capability evolved distinct and robust gut bacterial communities

**DOI:** 10.1093/ismeco/ycaf160

**Published:** 2025-09-11

**Authors:** Małgorzata M Lipowska, Edyta T Sadowska, Kevin D Kohl, Paweł Koteja

**Affiliations:** Institute of Environmental Sciences, Faculty of Biology, Jagiellonian University, 30-387 Kraków, Poland; Institute of Environmental Sciences, Faculty of Biology, Jagiellonian University, 30-387 Kraków, Poland; Department of Biological Sciences, University of Pittsburgh, Pittsburgh, PA 15260, United States; Institute of Environmental Sciences, Faculty of Biology, Jagiellonian University, 30-387 Kraków, Poland

**Keywords:** artificial selection, herbivory, microbiome, hologenome, cohabitation, rodents

## Abstract

According to the “hologenome” theory of evolution, natural selection and evolution can act through a conglomerate biological unit, the “holobiont”—the host and its associated microbiome. Although the concept is appealing and emerges as a unifying paradigm, its merits are debated, and few attempts have been made to directly test its specific assumptions using the approaches of experimental evolution. Here, we fill this gap using a unique model system: lines of bank vole (*Clethrionomys = Myodes glareolus*) selected for enhanced ability to grow or maintain body mass in 4-day test with a low-quality herbivorous diet and unselected control lines. Results from a complex nature–nurture design, in which we combined the selection experiment with dietary treatment and cohabitation between individuals from the distinct lines (to allow for horizontal bacterial transfer), showed that the “herbivorous” voles harbored a cecal microbiome community with altered membership and structure, and altered abundances of several phyla and genera, regardless of the origin of the cohabitant. Although the differences were small, they were statistically significant and partially robust to changes in diet and housing conditions. Microbial characteristics also correlated with host selection-related performance traits at the level of individual variation. These results, combined with those of a complementary cross-fostering experiment, showed that under these contexts, the microbiome is largely determined by genetic background (effect of selection) and early maternal effects, and can be altered in response to selection acting on other organismal traits. Such results are consistent with assumptions underlying the concept of hologenomic evolution.

## Introduction

Symbiotic associations between animals and the microbes they host (i.e. their “microbiome” or “microbiota”) have been increasingly recognized as crucial for the functioning of individuals at all levels of biological organization and the evolution of diverse morphophysiological adaptations [1–6]. This has led to the development of the “hologenome” theory of evolution, which argues that natural selection and evolution can act through a conglomerate biological unit of the “holobiont,” i.e. the hosts along with their microbiome, and hence modify the whole “hologenome” (genes of the host and of the microbiota) [7, 8]. The concept is emerging as a unifying paradigm [9], yet its interpretation and usefulness in understanding evolution is subject to a debate [8, 10–16]. Even the meaning of the term “holobiont,” first proposed in the 1900s, has fluctuated over time, and can be understood in several ways, ranging from seemingly neutral interactions without implicated “unity” or “integration,” to tight co-evolutionary host–microbe relationships [10, 16]. Here, we understand a “holobiont” to be a host with its microbiome, whose composition is at least partly controlled by the host regulatory mechanisms, a set of interactions sometimes described as an “ecosystem on a leash” [14, 17]. So far, only a few attempts have been undertaken to test specific assumptions or predictions of the hologenome theory of evolution directly using the experimental evolution approach [18, 19]. Here, we fill this gap using an experimental model of the evolution of herbivory in rodents.

Controlled laboratory experiments, particularly those with germ-free rodents, provided insight into specific effects of particular bacteria and mechanisms of their function [20–22]. However, the question how the beneficial “host–gut microbiota” associations might coevolve remains open [18, 23]. Experimental evolution bridges the gap between comparative and phenotypic-manipulation studies, and allows testing hypotheses concerning micro-evolutionary processes and the underlying mechanisms, from molecular to behavioral [24]. This approach has been underutilized in the research on gut microbiota [18], but recently several experiments have shown that applying a selective regime to the host can lead to changes in host microbiome, observed as differences in the microbiome community composition between the selected and control lines, or between divergently selected lines [19, 25–34].

To our knowledge, no previous selection experiment has simultaneously documented the stability of differentiated gut microbiomes with respect to dietary variation and potential microbial exchange with other individuals, and the beneficial role of the microbial differences with respect to the selected trait. A study of rats divergently selected for saccharine preference showed that the lines had distinct microbiomes that were mostly maintained despite cohabitation, suggesting stability of the microbial alteration [35]. However, a functional role of that difference in relation to the trait under selection was not considered. On the other hand, in two experiments on fish selected for mass gain, fish from the selected and control lines hosted different microbiomes regardless of diet [36, 37], but microbial exchange was not allowed.

Here, we employed an ongoing selection experiment on a nonlaboratory omnivorous rodent, the bank vole (*Clethrionomys = Myodes glareolus* Schreber 1780), comprising four random-bred control (C) lines and four “herbivorous” lines (H) selected for an improved coping with a low-quality diet (LQD; Supplementary Methods and Supplementary Fig. S1) [38–41]. This selection experiment is a particularly suitable model to test the concept of hologenomic evolution because herbivory is a complex adaptive strategy that requires a partnership between hosts and microbes [42–44]. In generation 15, the H-line voles had a modified bacterial community inhabiting the caecum and forestomach [19]. Although those individuals were fed a standard diet (SD) throughout their lives, their parents had experienced the special diet as juveniles, which could affect their offspring microbiome [45, 46]. Therefore, to distinguish the effects of selection *per se* (genetic differences) from the possible carry-over effect through vertical bacterial transfer (maternal environment effect), in the next experiment we analyzed the microbiome of voles reared by parents never exposed to the special diet, and the experimental evolution was combined with cross-fostering and diet manipulation [47]. The results showed that the evolved differences in cecal microbiome were partially robust to the vertical bacterial transfer from foster mothers and to dietary changes. Importantly, several of the microbial characteristics were correlated with the selection-related physiological traits, i.e. the evolved microbiome was beneficial (adaptive) in this experimental evolution model.

**Figure 1:**
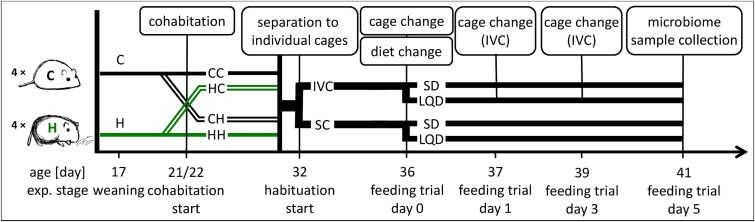
Scheme of the cohabitation experiment on voles from the selection experiment. Animals were sampled from four replicate “control” (C) and four replicate “herbivorous” (H) lines, and cohoused in pairs of individuals from either the same selection linetypes (CC and HH) or different linetypes (CH and HC). Feeding trials were then conducted in two types of cages (SC, standard cages; IVC, individually ventilated metabolic cages), with two types of diet (SD, standard diet; LQD, low-quality diet), and finally cecal samples were collected. (cartoons: January Weiner)

Here, we tested the same general hypotheses as in [47], allowing for tests of reproducibility (see [48, 49]), while also asking novel questions under unique contexts to test the robustness of the altered microbiome to horizontal bacterial transfer in juveniles (Fig. 1). Recent work has shown significant transmission of microbes along social networks, which can eventually overrun the effect of maternal transmission, as it occurred in adult wood mice [50]. As bank vole juveniles are highly mobile, the question about robustness of the microbiome to horizontal transfer in juveniles is particularly interesting. Therefore, we cohabited young independent animals from the same or alternative selection linetypes before measuring selection performance traits and analyzing cecal bacterial composition (Fig. 1).

According to the concept of hologenomic evolution, a selection toward an increased performance in a trait depending on the microbial symbiont function should result not only in an adaptively altered microbiome composition, but also in the host’s ability to maintain the altered microbiome under variable environmental conditions and when exposed to the possibility of horizontal transfer from individuals hosting a different microbiome. If this assumption is true, within our experimental evolution model, then the microbiome characteristics should differ between H and C lines (i) regardless of diet and (ii) regardless of the linetype of the cohabitant. Additionally, if the microbial differences have functional effects on the selected-related trait, then (iii) some of the microbiome characteristics should correlate with these performance traits at the level of individual variation. Conversely, if the evolved microbiome is not robustly retained, but instead labile and can be easily altered by horizontal transmission, then (iv) both the microbial composition and the performance traits should depend also on the linetype of the cohabitant. Verification of these hypotheses will provide clarity toward processes of hologenomic evolution, particularly whether the microbiomes modified as a result of the selection are labile and can be easily exchanged across cohabitants, or whether the host regulates the microbial composition as an “ecosystem on a leash” [17], which in turn affects the host performance [8, 33, 51].

## Materials and methods

### The animal model

We used bank voles (*Clethrionomys = M. glareolus*) from generation 27 of an artificial selection experiment comprising four replicate “H” lines, selected for an improved capability of juveniles to grow or maintain body mass in a 4-day test on an LQD, and four unselected C lines (Supplementary Fig. S1). The experiment was designed to mimic an evolutionary scenario in which omnivores such as the bank vole, which depend largely on condensed foods such as seeds and insects, face occasional shortages of such foods, and natural selection favors those individuals who can sustain or even grow on a strictly herbivorous, high-fiber diet for a period of time. The rationale and protocol of the experiment was presented in previously published papers [40, 41, 52] and in the Supplementary Methods. The difference between the selected and control lines remained also after two generations of relaxed selection (Supplementary Fig. S1), which indicates that it has a genetic basis. This has been confirmed by genome-wide SNP analysis (preliminary results: [53]).

The animals were maintained at constant temperature (20 ± 1°C) and photoperiod (16:8 light:dark), and, except for the selection trial, were fed a standard rodent chow: 23.9% protein, 4.5% fat, 5.3% fiber, and 14.3 kJ/g metabolizable energy in dry mass (Labofeed H, Kcynia, Poland).

### The cohabitation experiment

Details of the experimental design, measurement and analytical techniques, and statistical data analyses are presented in the Supplementary Methods. The experiment was performed on 828 males and females sampled from each of the four H lines and four C lines (Fig. 1). Parents of those animals were not subjected to the selection test. Information on the number of animals that completed subsequent stages of the experiment and reasons for exclusion are detailed in the Supplementary Methods and Supplementary Table S1.

At the age of 21–22 days, the cohoused pairs were formed from animals representing different linetypes (one C-line and one H-line animal; 229 pairs) or two animals from the same linetype, but different replicate lines (Supplementary Table S1). The pairs were placed in individually ventilated cages (IVC; AERO Mouse Green Line: Tecniplast, Bugugiatte, Italy), which prevented microbiome exchange with animals other than the cohabitant. Food and water were available *ad libitum*. Previous studies have shown that 7 days is enough for fecal transplants to stabilize [54] and the majority of microbial shifts introduced by co-habitation occur within the first 10 days [55]. Therefore, because we intended to perform the feeding trials (next step) at approximately the same age as in the selection experiment, we set the length of the cohabitation to 10 days.

At the age of 32 days, the pairs were split into individual cages and randomly assigned to four combinations of two factors: two categories of diet and two categories of cage type. The SD was the same as used in the regular maintenance (see above); the LQD was similar to that used in the H-line selection tests, but containing less plant material (pellets made of the mixture of 60% Labofeed H and 40% powdered dried grass: 20.4% protein, 4.4% fat, 16.1% fiber, and 11.4 kJ/g metabolizable energy in dry mass). The “standard” cage (SC) type was the same as applied during the H-line selection tests: open-top (model 1264C, Tecniplast, Bugugiatte, Italy), fitted with sawdust bedding. The IVC were the same as used in the cohabitation period, but fitted with “metabolic cage” type perforated plastic bottoms suspended above the cage floor instead of bedding, which allowed to collect all uneaten food and feces.

The animals were habituated to the new cages for 4 days (with SD diet *ad libitum*), and then the 5-day feeding trials were performed, in the same way as described in [47] (detailed also in Supplementary Methods). The rate of food consumption (FC, g/day) was measured as the difference between the dry mass of food provided and dry mass remaining in the cage. The rate of effective food digestion (FD g/day; a proxy for metabolizable energy intake) was calculated as a difference between the FC and feces production, and apparent digestive efficiency (ADE, %) as the FD/FC ratio. On Day 5, the animals were euthanized with isoflurane (Aerrane, Baxter, USA), and cecal contents were collected and stored at −80°C. For technical reasons, all the procedures on a given day were performed first on animals kept in the metabolic IVC cages (ca. 7:30 a.m.–11:30 a.m.h) and later on those in the SC (ca. 9:30 a.m.–13:30 p.m.h).

Animal welfare was monitored daily throughout the experiment. The procedures on animals were approved by the second Local Institutional Animal Care and Use Committee, Institute of Pharmacology PAN in Kraków (decisions 99/2017, 258/2017), in accordance with the EU directive 2010/63/EU.

### Microbial DNA analyses

The microbial DNA was obtained from 745 samples (Supplementary Table S1). The DNA was extracted, processed, sequenced, and analyzed as described in [47]. Details of the molecular laboratory and bioinformatic analyses (with references to the literature) are presented in the Supplementary Methods. Briefly, microbial DNA was extracted from the cecal samples with DNeasy Power Soil Pro kit (Qiagen, Germany), processed with a procedure targeting the V4 region of the 16S ribosomal RNA gene compatible with the Earth Microbiome Project, purified and indexed with a custom set of indexing primers. The indexed amplicons were pooled and sequenced by Novogene (UK) using the Illumina Novaseq PE250 technology. Approximately 23 000–148 000 (mean 55 000) raw read pairs per sample were obtained. The sequences were processed using the QIIME2 bioinformatic package [56–58]. After filtering out spurious and low-quality reads, the amplicon sequence variants (ASVs) were aligned and phylogenetic trees were constructed with the *phylogeny align-to-tree-mafft-fasttree* function. The taxonomic information of the ASVs was obtained with the feature-classifier *clarify-consensus-vsearch* tool and the *SILVA 138* database [59]. In the datasets and Supplementary tables with results, we retained phyla names such as recognized in this database, but in the text of Results we also give the names according to current nomenclature [60]. The sequences derived from mitochondria, chloroplasts, and archaea, and appearing in only one sample were excluded with the *feature-table filter-features* function. After all the steps of filtration, the mean number of reads per sample was ~28 000. The feature table was rarefied to the minimum of 13 874 reads per sample with the *feature-table rarefy* function. Twenty rarefied tables were generated for further bootstrap analyses.

The *diversity alpha* and *diversity alpha-phylogenetic* tools within QIIME2 were used on each of the rarefied tables to obtain five alpha-diversity metrics for 745 individuals: number of observed ASVs (N_ASV_), Shannon index, Shannon diversity (Shannon effective counts = exp(Shannon index)), Faith’s phylogenetic diversity, and Pielou evenness index. Weighted and unweighted UniFrac distance matrices were obtained for each of the rarefied tables with the *diversity beta-phylogenetic* tool, and a principal coordinate analysis (PCoA) plot was generated using the *diversity pcoa* function. Both the alpha-diversity metrics and the UniFrac matrices were averaged across the 20 repetitions to obtain the bootstrapped estimates. The whole bioinformatic pipeline code is presented together with the dataset in the pubic repository (see Data availability section).

Based on these initial results, we noticed 55 animals (7.4%) with strikingly low microbiome diversity (mean N_ASV_ = 130 vs 457 in the other voles), forming a separate cluster in both the heat map and beta diversity plots, but spread nearly evenly across the experimental groups (Supplementary Fig. S2). Detailed characteristic of this group is presented in the Supplementary Results. These individuals could be almost perfectly distinguished by a single criterion—the presence of bacteria from the [*Clostridium*] *innocuum* group. These voles also had lower body mass and food digestibility (see the Supplementary Results). Therefore, as those outlying individuals would distort the analyses of both the microbial and the physiological traits, we removed them from further investigation, leaving 690 individuals for the proper statistical analyses.

### Statistical analyses

The statistical analyses included four main parts, detailed in the Supplementary Methods. First, to test the effects of the experimental factors on body mass, the four feeding trial performance traits (MD_FT_, FC, ADE, and FD), and the three alpha-diversity metrics, cross-nested mixed ANCOVA models were fitted with SAS Mixed procedure (SAS v. 9.4 [61]). All the models were included the selection direction (linetype) of the focal individual and its cohabitant (H vs C lines), diet (SD vs LQD), and sex as the main fixed factors, interactions between these factors, and respective random effects. This model was further expanded adequately for specific analyses. The analyses of MD_FT_ and the microbial characteristics were performed both for all individuals with the cage type as a cofactor and separately for each of the cage types.

The effects of experimental factors on the multivariate beta-diversity of microbial community was tested with permutational multivariate analysis of variance (PERMANOVA) implemented in *adonis2* function in R (v4.3.0) *vegan* package (v2.6–4) [62, 63]. The analyses were performed for both the unweighted UniFrac distance matrix (the community membership) and the weighted UniFrac distance matrix (the community structure). The analyses were included the same fixed effects as described above, but were also performed separately for the diet and linetype subgroups.

To gain insight in what taxonomic groups contributed to the differences in the microbiome communities, we performed univariate analyses of abundances of 11 phyla, 115 genera, and 1344 ASVs present in >10% individuals. To avoid an excessive testing and problems with nonindependence of tests performed at different taxonomic levels, we limited the analyses to phyla (providing a broad perspective), genera (allowing to associate a particular taxon with a particular function), and to ASVs (providing insight into how particular bacterial strains within the genera respond to selection and other factors). The analyses were performed for relative abundances (using *adonis2* function), and for the “absolute” abundances, using the method of *Analysis of Compositions of Microbiome with Bias Correction* (ANCOM-BC; *ancombc2* function in R package ANCOMBC, v. 2.4.0; [64, 65]). The method corrects for the bias resulting from differences in sampling fractions among individuals, and allows to test the effects of both categorical and quantitative factors on the “absolute abundances,” and to estimate the effect sizes as log-fold changes (logs of ratios) of the abundances.

Finally, we analyzed the associations between the four organismal performance traits and the microbial characteristics at the level of individual phenotypic variation, by testing partial correlations in models including the same fixed predictors as in the main analyses. To assess the association with the overall microbial community membership and structure, we applied the same *adonis2* PERMANOVA models as described above, but with the performance traits and their interaction with diet included as additional predictors. Similarly, we used *ancombc2* to analyze the association with log-fold differences in “absolute” abundances of the particular taxa. The correlations with relative abundances were analyzed with linear models (R *lm* function), with the performance traits as the dependent variable, and the abundances as predictors.

The *P*-values obtained in the analyses of abundances were corrected using the Benjamini–Hochberg False Discovery Rate correction [66]. We assumed conventional *P* < .05 as the threshold of significance.

## Results

### Dominant microbiome taxa and alpha-diversity

In the 745 cecal samples, 5230 ASVs were identified, which were classified into 12 phyla, and 70 taxonomic families (Supplementary Tables S1–S3). The majority of ASVs (4659, 89.1%) were identifiable to 168 genera (143 with confirmed taxonomy). As we mentioned in Materials and methods section, 55 voles with a strikingly distinct bacterial community were excluded from the main analyses (details in the Supplementary Results). In the remaining 690 samples, for which the main analyses were performed, 5148 ASVs were classified into 11 phyla, 66 taxonomic families, and 144 genera (120 with confirmed taxonomy; Supplementary Tables S1–S3).

**Figure 2:**
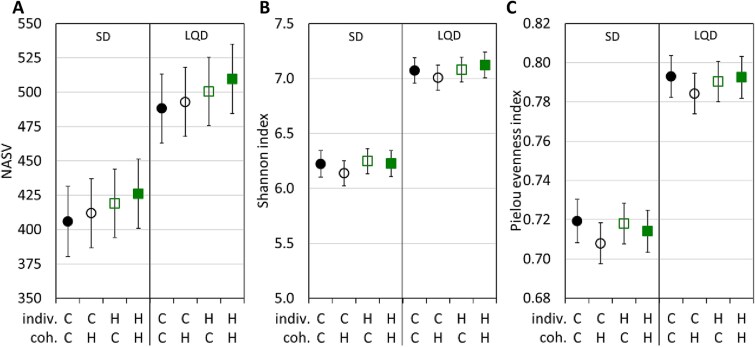
Alpha-diversity metrics of the cecal microbiome. (A) The number of amplicon sequence variants (N_ASV_), (B) Shannon index, and (C) Pielou index. The values are adjusted least-squares means (± 95% CI) from the mixed linear models estimated for the main experimental subgroups (diet: SD vs LQD diet; effect of the selection, H vs C: Indiv., selection linetype of the focal individual; coh., selection linetype of the cohabitant).

The number of ASVs (N_ASV_), Shannon index, Shannon diversity, Faith’s phylogenetic diversity index, and Pielou index of evenness, were higher in animals fed the LQD than those fed the SD (all *P* ≤ .0001; Fig.  2; Supplementary Tables S4 and S5). The selection linetype of the focal individual or its cohabitant did not significantly affect these indices (effect sizes were an order of magnitude smaller than those for diet; all *P* ≥ .15; Supplementary Table S5).

### The microbiome beta-diversity and abundance of particular taxa

**Figure 3:**
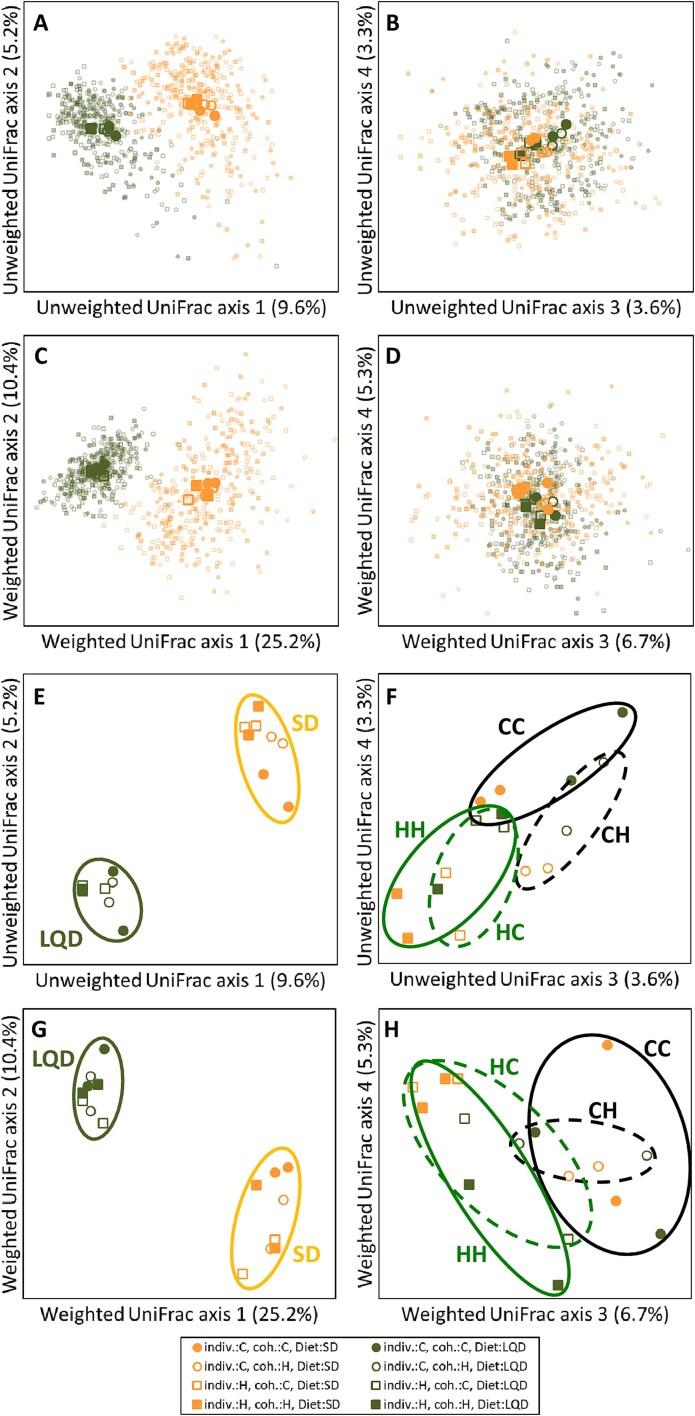
Caecum bacterial community beta-diversity characteristics. The values show scores on PCoA axes (left, axes 1 vs 2; right, axes 3 vs 4), based on UniFrac distances: (A, B, E, and F) unweighted (community membership), and (C, D, G, and H) weighted (community structure). (A–D) Individual data points with added centroids of the main experimental groups (larger symbols), (E–H) a close-up view of the centroids of the experimental groups (ovals are added for clarity of the information and have no statistical properties; all inferences are based on the results of PERMANOVA, presented separately).

Diet explained 8.1% of the joint variation in the bacterial community membership (unweighted UniFrac distances), and 24.9% variation in the community structure (weighted UniFrac; PERMANOVA, both *P* < .001; Supplementary Table S6), and dominated the first two PCoA axes (Fig.  3A, C, E, and  G). Sex, cage type, body mass, litter size, and date and time of sampling had only small effects on these community characteristics (≤0.3% of total variance).

Selection linetype of the focal individual explained 1.2% of the variation in community membership and 0.8% variation in community structure (both *P* < .001; Supplementary Table S6). These differences are represented on the third and fourth PCoA axes for the community membership, and on the third axis for the community structure (Fig.  3B, D, F, and  H), and were not modified by the origin of cohabitant (interactions *P* ≥ .2), but were more pronounced in animals fed SD than LQD (interaction: *P* ≤ .008). The cohabitant linetype only slightly affected the community membership (0.2% of variance, *P* = .044) and not the community structure (*P* = .6). The analyses split for diet and linetype groups confirmed that the focal individual linetype, but not cohabitant linetype, affected significantly the community membership and structure (Supplementary Table S6).

**Figure 4:**
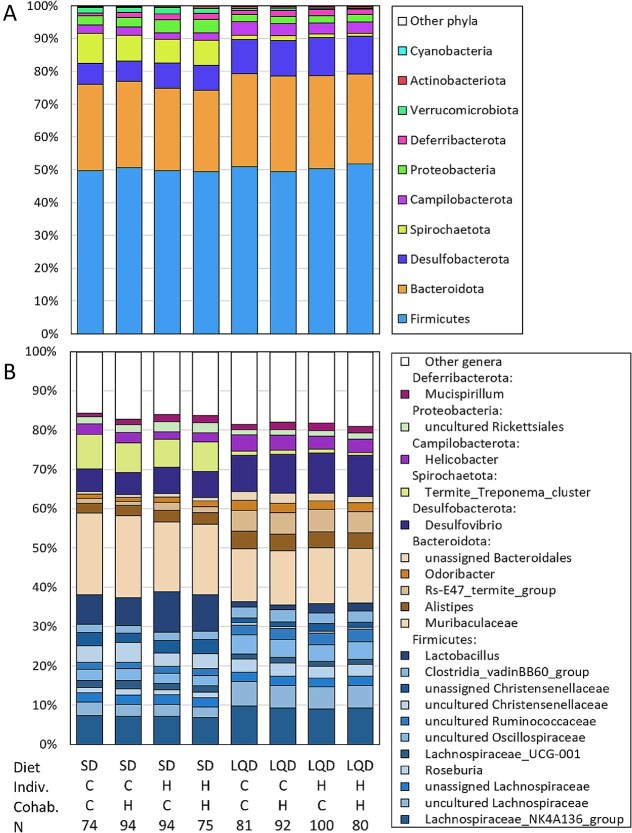
Cumulative relative abundance of the bacteria taxa. (A) Main bacterial phyla, (B) The most abundant and universal genera (abundance >1% of total DNA reads and present in >10% individuals). The abundances (cumulative %) are shown for the main experimental subgroups (diet: SD vs LQD diet; effect of the selection, H vs C: Indiv., selection linetype of the focal individual; coh., selection linetype of the cohabitant; N, sample size). The phyla names on the figure are such as in the *SILVA 138* database, but according to current nomenclature some have different names: Firmicutes, Bacillota; Desulfobacterota, Thermodesulfobacteriota; proteobacteria, Pseudomonadota; Actinobacteriota, Actinomycetota; cyanobacteria, Cyanobacteriota.

**Figure 5:**
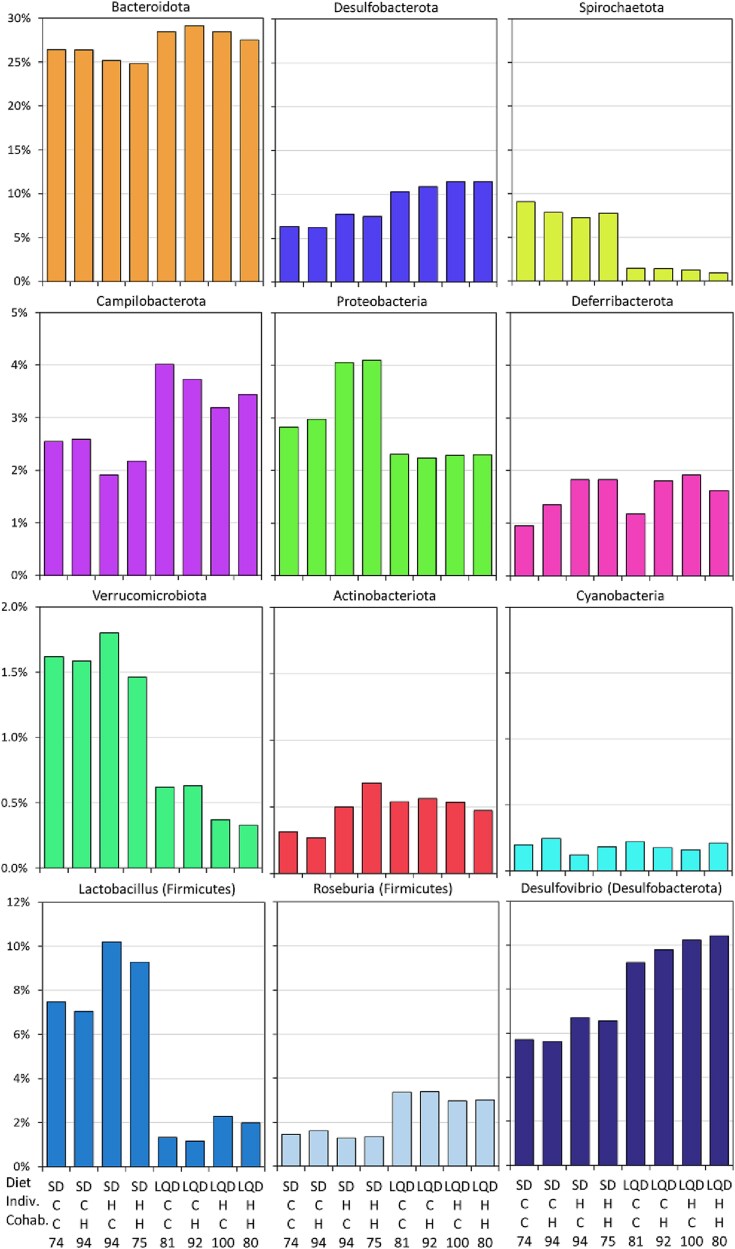
Relative abundance (%) of the bacteria taxa. Upper three rows: the main bacterial phyla (without firmicutes = Bacillota, whose abundances can be compared in Fig.  4); Bottom row: the examples of three genera in which the relative frequencies differed markedly between the selection linetypes. The abundances (%) are shown for the main experimental subgroups (diet: SD vs LQD diet; effect of the selection, H vs C: Indiv., selection linetype of the focal individual; coh., selection linetype of the cohabitant; N, sample size). The phyla names on the figure are such as in the *SILVA 138* database, but according to current nomenclature some have different names: Desulfobacterota, Thermodesulfobacteriota; proteobacteria, Pseudomonadota; Actinobacteriota, Actinomycetota; cyanobacteria, Cyanobacteriota. The figure does not show standard deviations or standard errors, because they might be mistakenly treated as a basis for inferring significance of the differences between the groups (the inferences were based on models including several variables, and the tests were based on the Monte Carlo approach; see Supplementary Tables S9 and S10).

We analyzed differential abundances of 11 phyla, 115 genera, and 1344 ASVs with univariate models using two metrics: untransformed relative abundances and bias-corrected absolute abundances (ANCOM-BC2) (Figs  4 and 5; Supplementary Results, Supplementary Tables R1 and R2, and Supplementary Tables S7–S14). Diet affected the relative and/or absolute abundances of all the phyla except Deferribacterota, and most of the genera and ASVs. Both methods showed that the LQD increased the abundances of Thermodesulfobacteriota [*SILVA 138* database name: Desulfobacterota] and the Candidate Phyla Radiation group [Patescibacteria], and decreased that of Spirochaetota, Pseudomonadota [Proteobacteria], and Verrucomicrobiota.

The selection line of hosts affected the abundances of several taxa and ASVs (Figs  4 and 5; Supplementary Results, Supplementary Tables R1 and R2, and Supplementary Tables S7–S14). Voles from the H lines, irrespective of the linetype of the cohabitant, had higher relative and/or absolute abundances of Pseudomonadota [Proteobacteria], Deferribacterota, Thermodesulfobacteriota [Desulfobacterota], and Actinomycetota [Actinobacteriota], but lower abundances of Bacteroidota, Campylobacterota, Verrucomicrobiota, and Cyanobacteriota [Cyanobacteria] (all *P* ≤ .057; Fig.  4A). The abundance of Bacillota [Firmicutes] did not differ between linetypes (*P* = .8), but the selection affected both the relative and absolute abundances of 8 its genera, only the relative abundance in 31 genera, and only absolute abundance in one genus (Figs  4B and Figure  5). For example, in the H lines the abundances of *Acetatifactor, Ruminococcus, Lactobacillus, Ileibacterium,* and *Anaeroplasma* were increased, and those of *Roseburia, Blautia,* and *Fournierella* were decreased. In other phyla, the selection affected both the relative and absolute abundances of 7 genera, only the relative abundance in 12 genera, and only absolute abundance in 2 genera. For example, the abundances of *Desulfovibrio*, *Mucispirillum*, *Bifidobacterium* and were increased in H lines, whereas those of *Rikinella*, *Muribaculaceae*, and *Bilophila* were decreased.

The cohabitant origin did not affect significantly the relative abundance of any of the phyla or genera, and affected the absolute abundances of only one low-abundance genus (Supplementary Tables S9, S10, S12, and  S13).

Abundances of several genera showed dependencies on interacting factors, particularly with diet type, and the effect of selection appeared significant with only one of the diets, or differed in magnitude, as in the case of relative abundance of *Lactobacillus,* where the difference was larger on the SD diet (Supplementary Table R1, Supplementary Tables S7, S10, and  S13).

Selection affected significantly the relative abundance of 427 ASVs (47% of those analyzed) and absolute abundance of 120 ASVs (26%; Supplementary Table R2, Supplementary Tables S8, S11, and  S14). In many cases where the abundance of a genus was not affected, the abundances of several of its ASVs differed in opposite directions between the H and C lines. Furthermore, in most genera for which abundances did not differ significantly between selection linetypes, abundances of some ASVs differed significantly in opposite directions. These opposite trends were also common within species (Supplementary Table R2). Interestingly, several ASVs were present nearly exclusively in the selected H lines. The most striking example of an ASV from an uncultured genus of Puniceicoccaceae (ITax = 5600 in Supplementary Table S8). This ASV was present in 42% of voles from the H lines, but in only three voles from the C lines (0.8%), all of which were cohabited with voles from the H lines. Conversely, the linetype origin of cohabitant did not significantly affect the relative abundance of any ASV, and affected the absolute abundance of only 27 ASVs.

### Performance in the feeding trial

**Figure 6:**
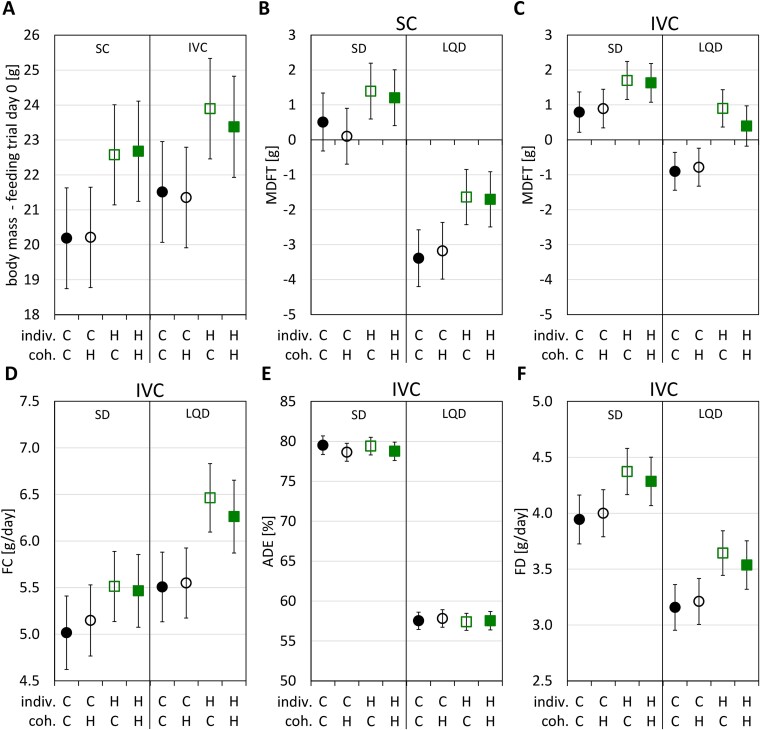
Performance in the feeding trial. (A) Initial body mass; (B and C) body mass change during the feeding trial (MD_FT_; divided by the cage type, standard SC vs individually ventilated metabolic cage); (D–F) FC rate, apparent digestive efficiency (ADE = 100 × FD/FC), and effective FD rate (FD = FC – Mass of feces/day) (these traits were measured only in the IVC metabolic cages). The values are adjusted least-squares means (± 95% CI) from the mixed linear models estimated for the main experimental subgroups (diet: SD vs LQD diet; effect of the selection, H vs C: Indiv., selection linetype of the focal individual; coh., selection linetype of the cohabitant). All these values (except initial body mass) were adjusted for the initial body mass and a few other covariates (see Statistical analyses subsection; details in Supplementary Methods).

The initial body mass was lower in animals moved to the SC than in those kept in the individually ventilated metabolic cages (*P* < .001; Fig.  6A; Supplementary Tables S4 and S5). This difference may be partly due to the fact that measurements were performed an average of 2 h later in animals kept in SC. The body mass change during the feeding trial (MD_FT_, g/5 days), the rate of FC (dry mass g/d), and the effective FD rate (dry mass g/d, a proxy for metabolizable nutrients and energy intake) increased with body mass, while ADE (%) decreased with mass (Supplementary Figs S3 and S4, Supplementary Table S5). All further results are provided as mass-adjusted values.

H-line voles gained more (or lost less) mass than C-line voles irrespective of the diet type, but the magnitude of the effect differed between diets and cage types (Fig.  6B and C; Supplementary Table S5). As expected, the ADE of LQD was much lower than that of SD, and despite a higher rate of consumption, animals fed LQD effectively digested less food (Fig.  6D–F; Supplementary Table S5). Voles from H lines consumed more food than those from C lines, and therefore, despite no difference in ADE, digested more food per day than the C-line ones (3.96 ± 0.18 g/d vs 3.58 ± 0.18 g/d; *P* = .006), irrespective of the food type (interaction: *P* = .7). None of the traits depended on the cohabitant origin (Fig.  6; Supplementary Table S5).

### Individual-level phenotypic partial correlations between host and microbial characteristics

Metrics of MD_FT_, FC, and FD were not consistently correlated with the alpha-diversity indices, but ADE increased with N_ASV_ (*P* < .001) and with Shannon index (*P* = .022; Supplementary Fig. S5A and B and Supplementary Table S15). However, MD_FT_, especially in animals fed LQD, was correlated with both of the beta-diversity metrics (Table 1). Both FC, ADE, and FD were correlated with the community membership, whereas the community structure was correlated only with ADE (Table 1).

**Table 1:** Results (*P*-values) of *adonis2* PERMANOVA analyses of partial correlation between physiological performance traits and two bacterial beta-diversity metrics (community membership and structure).

	**Community membership**				**Community structure**			
	**Both diet types**		**SD**	**LQD**	**Both diet types**		**SD**	**LQD**
	trait	trait × diet	trait	trait	trait	trait × diet	trait	trait
In both cage types								
MD_FT_	0.01	0.001	0.006	<0.001	0.06	0.046	0.311	<0.001
In SC								
MD_FT_	0.181	0.057	0.448	<0.001	0.085	0.091	0.406	<0.001
In IVC								
MD_FT_	0.022	0.034	0.019	0.004	0.25	0.149	0.367	0.021
FC	0.003	0.456	0.002	0.044	0.14	0.132	0.103	0.168
ADE	<0.001	0.072	<0.001	<0.001	0.014	0.061	0.001	0.014
FD	0.016	0.651	0.02	0.093	0.251	0.314	0.29	0.252

Notes: The community membership and structure are characterized by multivariate unweighted and weighted UniFrac distances, respectively. The analyses were performed with the same models as those testing the effects of selection and experimental factors on the beta-diversity metric, with three additional factors: a covariate representing a performance trait, its interaction with diet, and a covariate representing time of day at the start of the performance trait measurement. MD_FT_, body mass change in the feeding trial g/4d); FC, food consumption rate (g/d); ADE, apparent digestive efficiency (%); FD, the rate of effective food digestion (a proxy for metabolizable energy intake, g/d).

Metrics of MD_FT_ and ADE were correlated with both the relative or the absolute abundances of most bacterial phyla and many genera, but abundances of only a few genera were correlated with FC or FD (Supplementary Fig. S5, Supplementary Table R1, Supplementary Tables S15 and S16). As could have been expected, the correlations were often complicated by significant interactions with diet, and, in the case of MD_FT_, differed also between two types of cages. Detailed results of these analyses are presented in the Supplementary Results.

## Discussion

We have shown in our experimental model of the evolution of herbivory, that selection on host performance traits leads to the hosts maintaining a distinct microbiome, i.e. partially robust to dietary changes, correlates with host performance, and is robust to horizontal bacterial transfer from individuals harboring a different microbiome. Such results support the assumptions underlying the concept of hologenomic evolution outlined in Introduction section [8, 33, 51]. In particular, it seems that selection on the host may induce changes in aspects of host control of the microbiome [17].

Voles from the “H” lines were larger at the start of the feeding trial and grew faster on both diets during the trial than those from the C lines (Fig.  6). They also had a higher rate of digestion of the LQD, and thus an increased metabolizable energy intake, due to an increased rate of FC rather than increased digestive efficiency. However, the ability to consume and process the low-quality food at a higher rate without compromising digestive efficiency indicates an improved capacity for herbivory, given the usual tradeoff between digestion rate and digestive efficiency [67]. All these results were very similar to those obtained in the independent experiment involving cross-fostering instead of cohabitation [47], demonstrating integrity and repeatability of our experimental protocols.

As expected, the vole gut microbiome was strongly modulated by diet. The bacterial communities of voles fed the grass-diluted, fiber-rich LQD, were more diverse than those of voles fed the SD, as shown by increased values of all three alpha-diversity traits we analyzed. Voles from the LQD group had also a different community membership and structure, and different abundances of most of the bacterial taxa. These changes reflect previous observations regarding feeding on fibrous diets [68].

Our primary interest in this study, however, was in testing whether differences in the microbiome induced by our selection regime were labile and bacteria could be easily exchanged across cohabitants, or where robust due to a host control mechanism allowing to maintain these differences in the face of horizontal transfer. Both the bacterial community membership and structure differed markedly between the H and C lines (both *P* = .0001). Further, we showed that the microbiome community of juveniles is highly robust to horizontal transmission from other voles: the community membership was only marginally affected (*P* = .044), and community structure was not affected at all by the linetype of the cohabitant (*P* = .6; Supplementary Table S6; Fig.  3).

Interestingly, our previous study testing these hypotheses shows similar general patterns, but the effect of selection was more profound in the current experiment (compare Fig. 3 in [47] with Fig.  3 in this study). A plausible explanation is that in the cross-fostering experiment the difference attributed to selection was mainly due to genetic differences, whereas pups in the current experiment were reared by their biological mothers, which provided an opportunity for vertical bacterial transmission as well as physiological and behavioral early life maternal stimuli. The importance of early life vertical transmission from mothers in shaping the microbiome was documented by a highly significant effect of foster mother origin, albeit associated with different bacteria than those dependent on genetic background [47]. Thus, the difference between the microbiome of postweaning juveniles from the H and C lines is shaped both by the genetic and maternal environmental effects.

Importantly, both experiments showed that the bacterial taxa that exhibited differential abundance due to transmission, be it vertical (the selection linetype of the foster mother) or horizontal (linetype of cohabitant), had little overlap with the genera that exhibited differences due genetic background of the focal individual (selection linetype). Moreover, with a few exceptions, the horizontal transfer in juveniles observed in this study appeared in different bacteria than those acquired by vertical transfer in early life reported in the cross-fostering experiment [47]. Thus, even if the presence or abundance of some bacteria is susceptible to the horizontal transfer, the postweaning juveniles from the selected lines appear to maintain the beneficial microbial composition preferentially hosted due to both genetic (effect of selection) and early life maternal effects. The analyses of ASVs abundances showed the same pattern. Even within genera whose overall abundance did not appear affected by selection, many ASVs differed between voles from the H and C lines, but, few ASVs were affected by the linetype of cohabitant. This picture is consistent with the observation that the linetype of the cohabitant had no effect on any of the selection-related traits (Fig.  6).

Taken together, the results of the two experiments support the idea that, under the context of our experiment, microbiome community characteristics can be treated like other organismal traits: determined early in life by genetic and maternal influences, and later subject to acclimation to specific dietary and habitat conditions, but largely robust to random influences from other conspecifics at later life stages. Thus, the results supported some of the main assumptions of hologenomic evolution [8, 10, 11], particularly the evolution of host control mechanisms over the microbiome community [17], although results of these two experiments could not provide insights into the mechanisms of this control.

Lipowska *et al*. [47] reported that the effect of selection explained ~1% of the total variance in multivariate community characteristics, which might raise doubts about whether such a difference would be reproducible in an independent experiment (see [48, 49]). The results of this independent experiment confirm that the differences, although small, are real. The similarity of our results to other studies based on rodent selection experiments [25, 31, 32, 34] and the functional correlations between the microbial and selection-related performance traits suggest that such differences are biologically meaningful.

The gut microbiome can facilitate mammalian herbivory not only through digestive processes, such as cellulose digestion and nitrogen recycling [69], but also through regulation of metabolism and body mass balance [70–72] and feeding behavior [73–75]. In this study, we could not investigate such functions directly. However, we found that several microbial characteristics (alpha-diversity metrics and abundances of specific bacterial taxa) were independently correlated with the selection-related morpho-physiological traits at the level of individual phenotypic variation. Although only correlative, the results give some insights into the functional aspects of the changes in the microbiome that appeared as a correlated response to the selection in our experimental evolution model.

Typically, a greater taxonomic diversity yields higher functional diversity, which is beneficial toward degrading the complex fibers present in plant material [68]. Here, we observed a positive correlation between the digestive efficiency and cecal microbial species richness, measured as both the number of ASVs and Shannon index (Supplementary Tables R1 and S15; notably, in these two independent studies, the partial regression slopes were nearly the same, which again reinforces credibility of the results), although these alpha-diversity metrics do not differ between the selected and control lines in this study, nor in Lipowska *et al*. [47]. In this context, it seems counterintuitive that the correlations were stronger in animals fed SD than in those fed LQD (Supplementary Tables R1 and S15). However, animals were only fed the LQD for a short time (several days), in accordance with the assumptions and objectives of this selection experiment (Supplementary Methods; [40]). Thus, the microbiome in these animals may be under stress or transition, and thus exhibit greater interindividual variation [76]. Under longer feeding periods, microbiome may become better “optimized” to cope with such a diet, and show the functionality more consistently among individuals.

Results of analyses of correlation between the digestive efficiency and multivariate beta-diversity characteristics showed the same pattern, again, highly consistently in [47] and in this study. Both the community membership and community structure were correlated with digestive efficiency, and the former also with the rate of FC and digestion, and all these correlations were stronger in animas fed the SD than LQD diet (Table 1). These results clearly document the significance of the bacterial community composition in the digestion.

However, the correlation between the beta-diversity metrics and body mass-change during the feeding trials (MD_FT_) was much stronger in animals fed the LQD, i.e. under the conditions such as those in the proper selection trials, than in those fed SD (Table 1). Taken together, the results, although only correlative, suggest that the bacterial composition is an important factor affecting growth rate of the voles, at least partly independently of its effect on the FD. The results also imply that the changes in microbiome not related to FD contributed to the difference between H and C lines in the mass balance. The significance of the microbial contribution to our experimental evolution of “herbivory” is revealed by significant effects of the selection on the bacterial abundances, and correlations of the abundances with the selection-related traits for several bacterial taxa whose function in either the digestion or regulation of metabolism is evident or plausible (Figs Figure  4 and Figure  5; Supplementary Fig. S5, Supplementary Table R1, and Supplementary Tables S7–S16). Due to limited space, the discussion of these interesting results is presented only in the Supplementary Results.

To summarize, our results support the hypothesis that selection on a host performance trait leads to evolution of robust maintenance of an altered microbiome composition, which is beneficial in the context of the experimental evolution model. The microbiome community characteristics are determined early in life by genetic and maternal influences, and are later subject to acclimation to specific dietary and habitat conditions, but are largely robust to dispersal from other conspecifics at later life stages. Consequently, the results can be regarded as supporting major assumptions of hologenomic evolution, particularly the assumption that host regulates the microbial composition, which in turn affects the host performance [8, 17, 33, 51]. However, we recognize that our experiment had limitations, one of which was that it focused only on bacteria and their abundance. Further research should include other components of the holobiont microorganisms, such as fungi and viruses, and include more advanced functional analyses (see [77]). Also, several questions remain open, such as what would be the outcome of the experiment if it were performed on adults, with a “mature” microbiome, what specific genetic changes in the H lines confer the altered microbiome, in what specific way the beneficial bacteria interact with the host through the lower-level mechanisms (molecular, biochemical, and neurobiological), and whether the microbiome in the selected lines evolved only by modifying the composition of bacteria from an available diversity, or whether specific genetically divergent bacteria evolved in response to the (hypothetical) hologenomic evolution. We believe continued work with our bank vole experimental evolution model system can answer such questions, and thus contribute to the understanding of hologenomic evolution of mammalian herbivory, and encourage the development of other similar experimental evolution approaches.

## Supplementary Material

Lipowska_Suppl_MethResFigsTablesR1-2_ycaf160

Lipowska_Suppl_TablesS1-S16_ycaf160

Supporting_information_ycaf160

## Data Availability

All the data used in the study are deposited in the RODBUK public repository (compliant with the Open Access Infrastructure for Research in Europe (OpenAIRE) guidelines): https://doi.org/10.57903/UJ/I9FVZ1. The bioinformatic and statistical code used for the analyses is available temporarily at: MicroCH_Analyses_Code.zip. and will be deposited in the RODBUK public repository upon the paper acceptance.
