# Supplementary material for: Experimental evolution of a mammalian holobiont: bank voles selected for herbivorous capability evolved distinct and robust gut bacterial communities
Source: ISME Commun. 2025 Sep 11;5(1):ycaf160. doi: 10.1093/ismeco/ycaf160 (PMC12516954; doi:10.1093/ismeco/ycaf160)
Supplement: Lipowska_Suppl_MethResFigsTablesR1-2_ycaf160 [file lipowska_suppl_methresfigstablesr1-2_ycaf160.pdf]

Supplementary Methods, Results and Figures to:

Experimental evolution of a mammalian holobiont: bank voles selected for herbivorous capability evolved distinct and robust gut bacterial communities

Małgorzata M. Lipowska, Edyta T. Sadowska, Kevin D. Kohl, Paweł Koteja*
pawel.koteja@uj.edu.pl

1. Supplementary Methods

1.1. Animal model and the ongoing selection experiment

This work was performed on bank voles (*Clethrionomys = Myodes glareolus* Schreber 1780) from generation 27 of an ongoing artificial selection experiment maintained at the Jagiellonian University (Poland). The experiment was designed to mimic an evolutionary scenario in which omnivores such as the bank vole, which depend largely on condensed foods such as seeds and insects, face occasional shortages of such foods, and natural selection favors those individuals who can sustain or even grow on a strictly herbivorous, high-fiber diet for a period of time. The rationale, history and protocols of the ongoing experiment were presented in earlier papers (Sadowska et al. 2008, 2015; Lipowska et al. 2020). Briefly, the colony was established with about 320 wild voles captured in 2000 and 2001. After 5-6 generations of random breeding, the selection experiment has been started, with "Herbivorous" (H) lines selected for the ability to maintain body mass during a 4-day trial, during which the young, growing animals are fed a low-quality diet, "diluted" with dried grass powder (see below for justification of assuming such a selection criterion). Four replicate H lines, and four unselected Control (C) lines are maintained to allow valid tests of the effects of selection (Henderson 1997), with 15–20 reproducing families in each of the 8 lines (to avoid excessive inbreeding). As average litter size in the voles is only about 4.5, up to three subsequent litters from each family are reared to provide enough animals for an effective selection. The animals are weaned at the age of 17 days (at day 18 a next litter can be born) and kept initially in family groups. At the age of 32-36 days the 4-day selection trial is performed on most animals from the selected lines (except individuals assigned to separate experiments) and a sample of individuals from the Control lines. The selection criterion is body mass change during the trial adjusted for body mass at weaning and body mass gain between the weening and the start of the trial (i.e., a residual of regression on the two covariates). The adjustment is made to avoid selecting for just a high or low values of body mass or growth rate. The selection is performed mostly within-families, i.e., from each full-sib family 1-2 males and 1-2 females with the highest scores are chosen for reproduction. However, when more than 17 families are available, the families in which all individuals have below-average scores (residuals lower than zero) are excluded from reproduction. If the best animals from the family fail to reproduce, next ones (if available) are selected.

Over the course of the selection experiment, the composition of the low-quality diet has been modified a few times in attempts to ensure that it poses a challenge, but not an overly severe one (Sadowska et al. 2015). This, together with the fact that the composition of the grass powder changed across time and seasonal changes (despite controlled thermal and light conditions), leads to considerable variation in the selected trait values across generations (Fig. S1). Nevertheless, already since generation 3, the H-line animals have been consistently able to maintain a more positive body mass balance during the trial when compared to the C-line ones, and despite the large among-generation variation, the difference in body mass balance between the selected and control lines was about 1.5-2.0g, corresponding to 1-2 units of phenotypic standard deviation. In generation 25, the last

in which the selection was performed, voles from the H lines have gained during the test 1.55±0.97g (mean±SD from pooled observations from the four replicate lines, 7.4% of the initial body mass), whereas those from C line gained only 0.10±0.89g (0.5% of the initial body mass). In generations 16-17, and in two generations (25-26) preceding the experiment reported here the selection was relaxed, and the regular tests with low-quality diet were not performed.

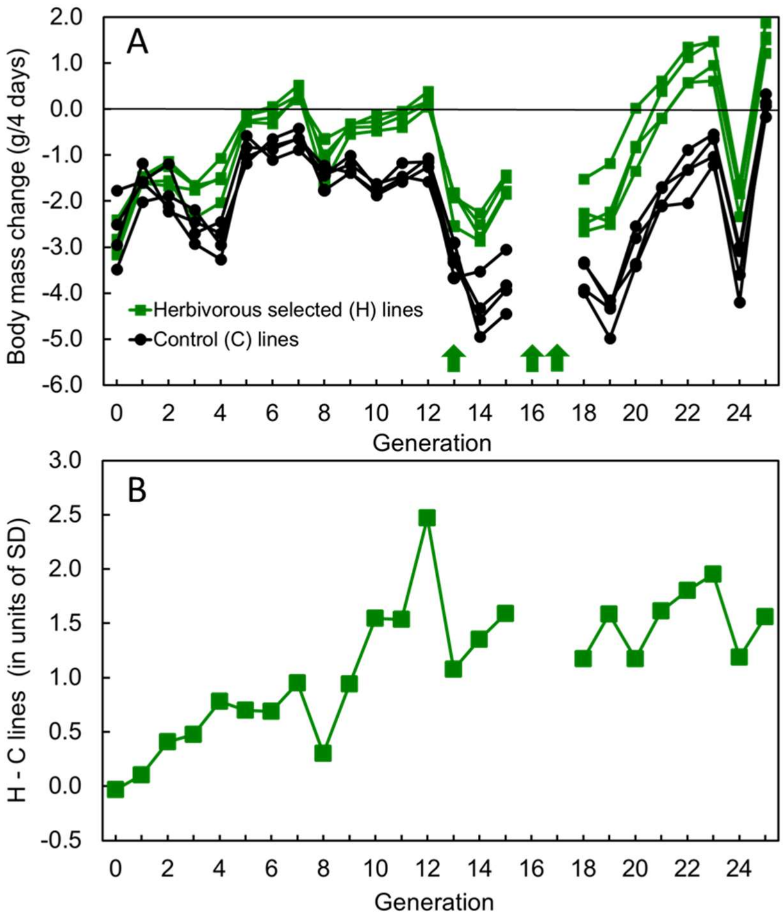

Fig. S1 Direct effects of selection towards an increased ability to maintain body mass in a 4-day test with low-quality herbivorous diet. **A)** Mean values of body mass change (g/4days) in the four replicate H-selected and four replicate Control lines; **B)** the difference between means of the selected and control lines in the units of phenotypic standard deviation; arrows indicate generations in which selection was relaxed.

Reproductive pairs were kept in standard polypropylene mouse cages (model 1290D, Tecniplast, Bugugiatte, Italy) fitted with sawdust bedding, a clay pot “shelter” and nest-building material (paper towel and a cardboard tube), with *ad libitum* access to water and food (a standard rodent chow: 23.9% protein, 4.5% fat, 5.3% fiber, 14.3 kJ/g metabolizable energy in dry mass; Labofeed H, Kcynia, Poland), at constant temperature (20±1°C) and photoperiod (16:8 light:dark; light phase starting at 02:00 hours).

All the breeding, selection and experimental procedures were approved by the Local Ethical Committees in Krakow, Poland (decision no. 170/2014 – 1st Local Ethical Committee for Animal Experiments, Faculty of Pharmacy, Jagiellonian University Medical College in Kraków; 257/2017 – 2nd Local Institutional Animal Care and Use Committee, Institute of Pharmacology Polish Academy of Sciences in Kraków), and in accordance with the EU directive 2010/63/EU. This study is reported in accordance with ARRIVE guidelines.

1.2. Justification of the selection criterion

The experimental evolution model was designed to mimic the early stages of the evolution of herbivorous strategy in mammals (Sadowska et al. 2008), a transition requiring symbiosis with bacteria digesting cellulose and other fibrous compounds of plant cells. Therefore, it could be argued that the digestibility of a herbivorous diet would be the appropriate target of selection. However, from an organismal and evolutionary perspective, coping with a particular diet in terms of percent digested mass may be less important than the ability to convert food into body growth or offspring. Therefore, we argue that the ability of juveniles to grow or maintain body mass during a short period of feeding on the low-quality diet (LQD) is an appropriate proxy for measuring "adaptation" to the herbivorous strategy. It is also consistent with the intended evolutionary scenario in which a non-strict herbivore may face a temporal shortage of typical food, and natural selection would favor those individuals that can immediately cope with the herbivorous diet (Sadowska et al. 2008, 2015). An important advantage of selection experiments is the potential to reveal the multi-level nature of phenotypic changes and to uncover proximate mechanisms that underlie the differences observed at the organismal level. For example, voles from the H lines tended to have a decreased basal metabolic rate, locomotor activity, and hormonal recovery after an acute stress (Sadowska et al. 2015; Maiti et al. 2019; Lipowska et al. 2020), but increased fat content (unpublished data). In this experiment, as in the parallel experiment based on the same model (Lipowska et al. 2024), we added microbiome as another level of investigation, and, in agreement with predictions based on comparative analyses, we showed that changes in microbiome are fundamental in evolutionary adaptation towards herbivory, and appear already at its initial stage.

1.3. The cohabitation procedure

We conducted a cohabitation experiment to allow for microbial transfer between alternate lines to test for microbial dispersal and effects on host phenotypes. The experiment was performed on animals from 69 C-line families and 80 H-line families from generation 27 (15-20 families within each replicate line, up to 7 siblings within family) (Fig. 1 in the main text). Parents of these animals were not subjected to the selection test. Because the first litter of a breeding couple usually differs from the further litters, the animals chosen for the experiment were sampled from 2nd and 3rd litters of each family. At the age of 17 days, the pups were weaned, weighed in clean cups, marked temporarily by fur clipping and moved, in groups of up to 5 siblings per cage, to standard housing cages (model 1264C, Tecniplast, Bugugiatte, Italy). The pups were provided sawdust bedding and a nesting material (a paper towel), and *ad libitum* access to food and water.

At the age of 21 or 22 days, experimental animals were separated from their siblings and moved to individually-ventilated cages (AERO Mouse IVC Green Line: Tecniplast, Italy), which prevented microbiome exchange with animals other than the cohabitant, fitted with sawdust bedding *and ad libitum* access to food and water. There, the animals were housed with another, unrelated experimental individual of same sex, forming a cohabitation pair. The pairs were formed with two individuals derived from either different line types (CH, HC – where the first letter denotes the linetype of the focal individual, and the second letter denotes the cohabitant linetype), or two animals from the same line type (CC, HH), but not the same replicate line (Fig. 1). At least one animal within a pair was 22 days old, animals a day younger were used if age-matched cohabitants were not available. If necessary, marking by additional fur clipping was applied to ensure distinction between cohabitants. Whenever possible, same-sex siblings were assigned to different replicate-line combinations. Within

each combination of replicate lines, 13-19 pairs were formed (414 pairs = 828 individuals total). The cohabitant pairs were housed together for 10 days, after which the animals were separated into individual cages. In cases where one animal was younger than the other, it was maintained in the cohabitation cage for an additional day.

One pair was incidentally killed during cohabitation. In two more pairs one of the cohabitants had died. In one of these cases bite marks were found on the corpse, indicating that the surviving animal had altered its diet and hence it was excluded from the experiment. In the other case the carcass was found fresh and intact at the day of separation, so the surviving animal was maintained in the experiment. Hence, 823 animals successfully completed cohabitation (see Table S1 for a summary of the number of animals that completed subsequent stages of the experiment).

1.4. The feeding trial

Next, we conducted a factorial experiment where animals from each cohabitation combination were fed two different diets. The animals were separated from their cohabitants at the age of 32 days and moved to individual cages where they were subjected to a feeding trial. The animals were assigned to four combinations of two factors: two categories of diet and two categories of cage type. The assignment was randomized, with a restriction that cohabitants were assigned to different cage type categories, and same-sex siblings were distributed possibly evenly across cage and diet type combinations. The two diet groups received during the feeding trial either the standard diet (SD), the same as used in the regular maintenance and breeding (see above), or low-quality diet (LQD), similar to that used in the H-line selection tests, but containing less plant material (pellets made of the mixture of 60% Labofeed H and 40% powdered dried grass: 20.4% protein, 4.4% fat, 16.1% fiber, 11.4 kJ/g metabolizable energy in dry mass).

The “standard” cage type (SC) was the same as applied during the H-line selection test: standard open-top cages (model 1264C, Tecniplast, Bugugiatte, Italy), fitted with sawdust bedding. The “metabolic” cage type (IVC) were individually-ventilated cages (AERO Mouse IVC Green Line: Tecniplast, Italy), the same as used in the post-weaning period, but fitted with perforated plastic bottoms suspended above the cage floor instead of bedding, which allowed to collect all uneaten food and feces (blotting paper was placed at the floor to absorb urine). The two types of cages were applied because reliable estimates of food consumption and digestibility require using metabolic cages, but on the other hand the lack of bedding in such cages is stressful, and therefore both the pattern of body mass changes and the microbiome composition could differ from those in voles maintained in standard cages with bedding.

The animals were habituated to the cages for four days. During this period, they were provided *ad libitum* the standard food in the feeder, but also offered a small pellet of the experimental diet (either SD or LQD, depending on the diet group assignation) on the cage bottom. The LQD was introduced in the habituation phase to minimize the effect of novelty at the onset of the proper trial.

After the 4-day habituation, at the age of 36 days, the 5-day feeding trial was started (day 0; Fig. 1). For technical reasons, all the procedures on a given day were performed first on animals kept in the metabolic IVC cages (ca. 7:30 - 11:30 hours) and later on those in the standard cages (ca 9:30 - 13:30 hours). To minimize the effect of the measurement timing on the estimates of body mass changes and food consumption, in the subsequent stages of the feeding trial the procedures were performed on animals ordered in the same way. At day 0, the animals were weighed in clean cups, moved to fresh

cages (of the same type), and were given either SD or LQD. In the standard cages, the food was provided in excess to the overhead feeder, as in the routine breeding or standard selection tests. In the metabolic cages, a pre-weighed portion of ca 12g food (weighted to the nearest 0.001g) was served on the cage bottom (to obtain reliable estimates of the food consumption the amount of food provided must be small, and with the small amount of food pellets some animals had problems with eating it from the feeders – 28 animals which originally received food to the feeders were excluded from further analyses). At the same time weighted samples of the food were taken for measuring dry mass content. At days 1 and 3 the animals were weighed in clean cups and either returned to the same cage (standard cages group) or moved to fresh cages with a pre-weighed, 23-g portion of food (metabolic cages). Although the food portions were designed to be more than sufficient for 2-day periods (days 1-3 and 3-5), some animals tended to grind it and the orts fell below the perforated floors. Therefore, the amount of food available was inspected every day, and was restocked if needed. Nevertheless, some animals had ground all available food to orts and temporarily lost access to food as it fell through the floor, and those 22 animals were excluded from further analyses.

Uneaten food and feces collected from the metabolic cages were pre-dried, sorted, dried (two days at +60°C in vacuum drier) and weighed (to the nearest 0.001g), alongside with the samples of food taken for dry-mass content estimation. The rate of food consumption (FC, g/day) was calculated for days 1-5 as the difference between the dry mass of food provided and dry mass remaining in the cage, averaged over the 4 days. For the same four days, the rate of food digestion (FD g/day) was calculated as a difference between the food consumption and feces production, and apparent digestive efficiency (ADE, %) was calculated as the FD/FC ratio. The first day of the trial (days 0-1) was not included in these calculations because the feces excreted for at least several initial hours were remains of the pre-trial food, and therefore the estimations of digestibility would be meaningless.

On day 5 the animals were moved to a separate room, weighed, euthanized with isoflurane (Aerrane, Baxter, USA) and dissected using flame-sterilized tools. The caecum was extracted, cut just before the entrance to ileum, and its contents were transferred to a clean Eppendorf tube. The tubes were immediately put on dry ice and stored in -80˚C within 2.5 hours.

During the feeding trial, 20 animals died, 5 showed signs of poor health (sudden mass changes, unhealthy look, cage wetting and smell distinct to early symptoms of diabetes) and one got accidently exposed to external microbial sources (non-sterilized laboratory equipment). Additionally, as was mentioned earlier, 50 animals had difficulties in accessing food in the IVC cages: 28 animals had their food provided in a feeder, where it was less available than on the cage floor, and 22 animals tended to fragment all available food into orts which fell through the perforated floor, and might have experienced brief fasting before the daily cage checking. Overall, samples were collected from 747 individuals, and microbial DNA was successfully extracted from 745 samples.

It should be noted here that we did not analyze the bacterial composition in animals fed LQD for a long period of time, sufficient to establishing of a bacterial community "optimized" to cope with such a diet. Instead, in accordance with the assumptions and objectives of this selection experiment (Sadowska et al. 2008), we studied a response of the animals and their microbiota to a sudden change in diet lasting only five days. We are aware that the experiments would be further strengthened by including groups fed both the SD and the LQD for a longer time and to an older age, enough to stabilize the microbial composition after the dietary change. However, doubling the effort would not be feasible due to logistical limitations and, more importantly, would not be acceptable for ethical reasons, since the

negative body mass balance in the 5-day trial indicated that many individuals, especially those in the unselected C lines, would not be able to live on this low-quality diet for a long period of time.

1.5. Microbial DNA analyses

All the microbial DNA analyses were performed in exactly the same way as in the parallel experiment, presented in Lipowska et al. (2004). Microbial DNA was extracted with DNeasy Power Soil Pro kit (Qiagen, Germany), according to the producer recommendation (Goller 2023), with a minimal modification: homogenization was performed with Bead Ruptor Elite (Omni International, USA) following a 2×30s 3.25m/s program with a 30s break). The tubes containing caecal contents were moved onto dry ice, and partially thawed on wet ice just before the DNA extraction. The contents of the tube were mixed with a flame-sterilized spatula, and a subsample of approximately 150mg was taken for the extraction. The extracts were further processed with a procedure targeting the V4 region of the 16S ribosomal RNA gene, compatible with the Earth Microbiome Project and utilizing a two-step PCR library preparation protocol (Method four in: (Glenn et al. 2019; Marquina et al. 2021)).

In the 22 cycles of the first PCR, the target region was amplified using custom 515F and 806R primers with variable-length inserts and Illumina adapter tails presented in (Iwaszkiewicz-Eggebrecht et al. 2023). The products were purified on home-made SPRI (sold phase reversible immobilization) magnetic beads and indexed in 7 cycles of the second PCR reaction using a custom set of 192 forward and 192 reverse indexing primers (Iwaszkiewicz-Eggebrecht et al. 2023). Each sample was indexed with a unique combination of two custom primers containing 10-b indexing sequences. Each of the primers was used in no more than 8 combinations, to allow for efficient recognition and exclusion of chimeric reads.

The indexed amplicons were pooled and sequenced by Novogene (UK) using the Illumina Novaseq PE250 technology. The samples were processed alongside with ones from a parallel experiment (Lipowska et al., 2024), as well as 49 negative control samples collected at various levels of sample extraction and processing procedure, and positive control samples created by duplicated DNA extraction and processing procedures performed on samples from 16 individuals. We ordered 50,000 raw read pairs per sample. However, because the negative controls had nearly no reads, the effective number of reads per sample was higher. For the samples from this experiment approximately 23,000-148,000 reads per sample were obtained (mean 55,000). Duplicated samples from 16 voles produced comparable results, which were then combined (188,000 – 251,000 reads per sample) before further processing. Negative control samples had much lower raw read count than regular samples (2-16,036 reads, mean 535).

The sequences were processed using the Qiime2 bioinformatic package (Bolyen et al. 2019; Marizzoni et al. 2020; Prodan et al. 2020). The primers were trimmed off with the *cutadapt* tool, which also filtered out the sequences in which the error rate within the primer region exceeded 10% (2 bases per primer), or which were shorter than 200b after trimming. The sequence pairs were assembled using the *PEAR* tool (Zhang et al. 2014), with a minimum overlap of 15b, minimum quality threshold of 30, and maximum assembly length of 300b. The assembled reads were clustered into sequence variants with the *deblur denoise-16S* tool, based on reads with length limited to 252b. Each of these steps cleared the data base of spurious reads characterized by high error rate, low read quality or improbably low similarity to known taxa. The amplicon sequence variants (ASVs) were aligned and phylogenetic trees were constructed with the *phylogeny align-to-tree-mafft-fasttree* function. The taxonomic information of the sequence variants was obtained with the feature-classifier *clarify-*

consensus-vsearch tool and the *SILVA 138* database (Quast et al. 2013). In the datasets and supplementary tables with results we retained phyla names such as recognized in this database, but in the text of Results we give also the names according to current nomenclature (Oren and Garrity 2021). The sequences derived from mitochondria, chloroplasts and archaea were excluded from the list with the *feature-table filter-features* function; the same function was also used to remove sequences found in only one sample. After processing, the number of reads per sample ranged from 13,874 to 79,543 in majority of the samples (mean 28,000), and from 90,703 to 125,394 (mean 103,000) in the 16 pooled duplicated samples. In negative control samples, the number of processed reads ranged from 0 to 10,438 (mean 313), and they clustered separately from the vole samples on an exploratory unweighted UniFrac plot. We decided that this indicates low probability of bias or sample cross-contamination, and excluded the negative control samples further analyses. The feature table was rarefied to 13,874 reads per sample with the *feature-table rarefy* function, to match the number of reads in all samples to that in the sample with the smallest number. Twenty of such rarefied tables were generated for further bootstrap analyses.

The alpha- and beta-diversity measures of the microbiome composition were obtained with tools available within the Qiime2 package. The *diversity alpha* and *diversity alpha-phylogenetic* tools were used on each of the rarefied tables to obtain four alpha-diversity metrics in 745 individuals: number of observed ASVs (N_{ASV}), Shannon index, Shannon diversity (Shannon effective counts = exp(Shannon index)), Faith's phylogenetic diversity, and Pielou evenness index. The values were averaged across the twenty repetitions to obtain the bootstrapped alpha-diversity values for each sample. Similarly, weighted and unweighted UniFrac distance matrices were obtained for each of the rarefied tables with the *diversity beta-phylogenetic* tool, and the matrices were then averaged to form a single bootstrapped matrix for each metric. A PCoA analysis was performed on the matrices with the *diversity pcoa* function.

Based on these initial results, we noticed that a subset of 55 animals (7.4%) were characterized by strikingly low microbiome diversity, and formed a separate cluster both in the heatmap and the beta-diversity (PCoA axes) plots (supplementary Results, Fig. S2, below). The subset could be nearly perfectly distinguished by a single criterion: the presence of bacteria from an undescribed genus from *Clostridium innocuum* group (*Ci*), which did not appear in any other individuals. The *Ci*-present animals were distributed nearly evenly across all the experimental groups. As a consequence, all the quantitative traits describing microbiome were plagued by an extreme non-normality of the within-groups distribution, which precluded any meaningful statistical tests concerning the experimental factors. In addition, the Ci-present voles had also a lower body mass and lower food digestibility (supplementary Results). Therefore, because those 55 outlying individuals would distort the analyses of both the microbial and the physiological performance traits, we removed them from further investigation, leaving 690 individuals for the proper statistical analyses.

1.6. Statistical analyses

The statistical analyses included four main parts: a) univariate analyses of the physiological traits measured in the feeding trials and the bacterial alpha diversity indices; b) multivariate analyses of the bacterial community composition and structure; c) univariate analyses of abundances of particular phyla, genera, and ASVs; and d) multivariate and univariate analyses of correlations between the physiological and microbial traits.

a) Univariate analyses of the physiological traits and the bacterial alpha diversity indices

The effects of diet and the origin of the individual (the genetic component of the effect of selection) and of the cohabitant (environmental effect associated with the distinct selection lines) on body mass, performance traits in the feeding trial, and alpha-diversity characteristics of the caecal microbiome at the end of the trial, were performed with cross-nested mixed ANCOVA models, using Mixed procedure of SAS (v. 9.4, (SAS Institute Inc. 2011)), with REML method of estimation and variance components restricted to positive values. All the models included the selection direction (linetype) of the individual and its cohabitant (H *vs* C lines), diet (SD vs LQD) and sex as the main fixed factors, interactions between these main factors, and respective random effects of replicate line of both the individual and the cohabitant (nested within respective selection groups), and random interaction of the lines with diet and sex. This basic model structure was further expanded to accommodate additional factors and covariates adequate for specific analyses. The models also included the following covariates: body mass (g; except in the model with body mass as the dependent variables), litter size at weaning, measurement date (counted as number of days since the birth of the first animal), and time of measuring body mass and sampling the cecum contents (in Excel format: proportion of 24h). We considered also models including "batch number" for groups of samples that were either extracted, processed or pooled at the same day as additional random effects (as the batches did not overlap, the analyses had to be performed separately for each of the three kinds of batches). Although some variation among the batches was detected, the analyses gave the same outcome for the main factors as the models without those effects, and therefore the batch numbers were not included in the final models.

The response variables were body mass change during the feeding trial (MD_{FT}; g/5 days), food consumption (FC; g/day) and digestion rate (FD; g/day), apparent digestive efficiency (ADE; %), and five alpha diversity metrics: number of ASVs (N_{ASV}), Shannon index, Shannon diversity, Faith's phylogenetic diversity index, and Pielou evenness index. Analyses of MD_{FT} and the microbial traits (which were measured in two cage types) were performed both for all individuals with the cage type as a cofactor (SC *vs.* IVC) and separately for each of the cage types. All the analyses were performed in two versions: for all individuals with *Ci* presence as an additional cofactor, and separately for the main, *Ci*-free group.

Each of the above models included initially all first-order interactions among all the main fixed categorical factors, and, if applicable, also the second order interaction between the effects of individual's and cohabitant's origin and diet, and corresponding random interaction terms (interactions between replicate lines and the respective fixed factors). Then the models were step-wise reduced by removing non-significant interactions. However, interactions between the three focal factors, the origin of individual and its cohabitant and diet, were always retained in the final models.

The set of random effects included in the above models reflected the actual structure of the experimental design (with two levels of random nested effects and numerous interactions at the level of replicate lines), and corresponded in a minimalistic way to the set of fixed effects in the model. We realize that, despite the large sample size, the number of these random effects was too large to be effectively estimated. However, as it was not possible to determine *a priori* which subset of random effects would be estimable for a particular dependent variable (and the sets turned out to be different for different variables), we decided to keep the excessive set and let the SAS Mixed procedure find the best solution. In all the models the majority of random effects were fixed to zero and only a few positive variance components were estimated. Therefore, the models effectively provided the same

solution for the fixed effects as would be obtained in models not including the excessive random effects. Because we used the Satterthwaite's approximation of degrees of freedom (df), the excessive, fixed-to-zero random effects did not affect results of ANOVA F and t tests, either. Note, that with Satterthwaite's approximation the effective dfs are computed from a combination of the dfs of respective random grouping effects and residual term, weighted by variance contribution of the terms (SAS Institute Inc. 2011), and therefore the dfs can take non-integer values.

Several analyses revealed outlying individuals (absolute value of studentized residual ≥ 4.0). These individuals were excluded from analyses of one or more traits, but were retained in analyses of other traits, in which their residuals did not stand out. There was one such individual for body mass at the start of the trial, three for MD_{FT}, five for ADE (which were also excluded from analyses of FC and FD) and six for the alpha-diversity metrics. The exclusion of these individuals from respective analyses improved the normality of residual distribution and the model's goodness of fit (judged by the models' AIC values).

The experiment was designed to be balanced, and it nearly was for the main factors. However, the models described above had many predictors, including several interactions, covariates and cofactors. Therefore, we checked for possible collinearity among the predictors. Since such analyses cannot be performed within the SAS Mixed procedure framework, we used the SAS Reg procedure to fit fixed-effects multiple regression models to each of the response variables. These models included the same set of fixed predictors as the final model fitted with the Mixed procedure. We used the "vif" and "tol" options to compute the variance inflation factor and tolerance (the reciprocal of VIF) for each predictor and the "collin" option to obtain a detailed diagnosis based on eigenvalue analysis (condition index and proportions of variance explained). The analyses showed no serious collinearity (all VIF < 7, and for eigenvalues with condition index >30 not more than one predictor explained more than 50% variance), except for that resulting from the correlation between the categorical effect of cage type and the daytime of performing the measurements. This correlation was expected because the measurements were performed on average two hours later in the SC than in the IVC cages. Consequently, the effects of those two factors are partly confounded. However, those effects were not of primary interest and their collinearity had no bearing on the assessment of the focal factors.

Complete tables with group composition, descriptive statistics, results of the linear mixed models (significance of all the effects and adjusted least-squares means with confidence intervals, LSM±95%CI) are provided in Supplementary Tables gathered in Excel file. The adjusted least-squares means were computed based on the final models for mean values of the covariates (the same for all analyses: litter size = 5.5; body mass at the onset of the feeding trial = 21.96g).

b) multivariate analyses of the bacterial community composition and structure

To analyze the effects of the focal factors (the origin of the biological and foster mothers and diet) on the multivariate beta-diversity characteristic of the microbial community we used permutational multivariate analysis of variance (PERMANOVA, with 9999 permutations) implemented in R (v4.3.0) *vegan* package (v2.6-4; (Anderson 2017; Oksanen et al. 2022)). The analyses were performed for both the unweighted UniFrac distance matrix (describing the community membership) and the weighted UniFrac distance matrix (describing the community structure). The models included also sex and cage type as additional main effects, as well as the covariates present in the univariate ANCOVA models described above. Initial models included all first-order interactions and the second-order interaction between the effects of individual's and cohabitant's origin and diet type. Then the models were step-

wise reduced in the same way as the univariate models presented above. As the analyses showed significant interactions between the three focal factors, in the next steps the analyses were performed separately for the diet and individual's or cohabitant's origin subgroups. Although *adonis2* PERMANOVA can handle random effects (Anderson 2017; Oksanen et al. 2022), it cannot cope with unbalanced nested designs. Therefore, in these analyses the random effects of replicate lines were not included (c.f. (McNamara et al. 2021; Hanhimäki et al. 2022)).

c) univariate analyses of abundances of particular phyla, genera, and ASVs

To get an insight in what taxonomic groups contributed to the differences in the microbiome beta diversity between the experimental groups, we performed univariate analyses of the relative abundances of 11 phyla (we omitted Fusobacteriota, which were present practically only in the *Ci-present* group) and 115 genera that were preset in at least 10% of *Ci-free* individuals. To avoid an excessive number of tests and problems with non-independence of tests performed at different taxonomic levels, we limited the analyses to phyla, which provides a broad perspective, to genera, where a particular taxon can be associated with a particular function. In these analyses unclassified and uncultured bacteria from higher taxa were operationally treated as "genera". In addition, to find out how the selection and other factors affected abundances of particular bacterial strains, we performed the analyses for 1344 ASVs that were present in at least 10% of *Ci-free* individuals. The analyses for relative abundances were performed with the *adonis2* PERMANOVA function because the distributions of the abundances were non-normal (and for many taxa and ASVs severely zero-inflated), and hence the regular linear model could not be used. In these analyses the dependent variable was the abundance of a particular taxon, and the structure of the predictor variables was such as used in the multivariate model presented above. The analysis was performed for the Euclidean distance matrix, and therefore the analysis was equivalent to PERMANOVA on Bray-Curtis dissimilarity for relative abundances of the focal taxon and "all other taxa combined" (summing up to 1), or to PERMANOVA on the weighted UniFrac distance for such two operational "taxa" with the sum of the length of phylogenetic branches set to 1. On the other hand, such an analysis is also equivalent to the classical univariate linear model for the abundance of the focal taxon fitted with the least-squares method, except that the reported ANOVA F test of significance uses Monte Carlo F distributions (generated with permutations) instead of the theoretical F distribution based on normality assumption. P-values obtained in these analyses were corrected using False Discovery Rate correction for multiple comparisons (Benjamini and Hochberg 1995) ("BH" option in R function *p.adjust*).

The above analyses based on the relative abundance of particular taxa are conceptually compatible with the multivariate analyses based on the distances computed from the matrix of relative abundances of ASVs. However, the approach has a drawback: tests for particular taxa are not independent, because a higher abundance of a taxon implies a decreased abundance of others. Therefore, we applied also the ANCOM-BC method (*Analysis of Compositions of Microbiome with Bias Correction*; *ancombc2* function in R package ANCOMBC, v. 2.4.0; (Lin and Peddada 2020a, 2020b)), to compare the bias-corrected "absolute" abundances. The method corrects for the bias resulting from differences in sampling fractions among individuals, and fits log-linear models to the corrected abundances. It does not provide estimates of "absolute abundances" *per se*, but allows to test the effects of both categorical and quantitative factors on the "absolute abundances", and estimate the effect sizes as log-fold changes (logs of ratios) of the abundances. Thus, the comparisons of the abundances across groups concerns log-fold differences. The analysis is based on logs of counts of ASVs, and therefore zeroes must be converted to a small positive value ("pseudocount"). The

ancombc2 function automatically performs the analysis of sensitivity of the results to the choice of the pseudocount, by fitting the model for a range of such values; if the results depend grossly on the choice of the pseudocount value, the sensitivity test is scored as failed. These outcomes differ between particular factors in the model independently for a given taxon, so for a given taxon the tests of some factors may be successful and for others not. Compared with several other methods, ANCOM-BC shown in simulations the best performance, both in terms of controlling the bias and the False Discovery Rate, and maintaining a high power of detecting differences in bacterial composition (Lin and Peddada 2020a, 2020b). The analyses were performed for the same set of phyla, genera, and ASVs, and with the same factors included in the model, as in the analyses for relative abundances. As well, the BH correction for False Discovery Rate was applied, but only for the sets of p-values that positively passed the sensitivity test; all other are reported as “not available” (“NA” in the Supplementary Tables).

d) multivariate and univariate analyses of correlations between the physiological and microbial traits

The last part of the analyses was aimed at testing phenotypic correlations between the traits characterizing performance in the feeding trials (MD_{FT}, FC, FD, ADE) and microbial characteristics at the level of individual variation, within the groups of the main factors (i.e., partial correlations). To assess the association of the performance traits with the overall microbial community membership (unweighted UniFrac distances) and community structure (unweighted UniFrac distances), we applied the same *adonis2* PERMANOVA models as described above, but with the performance traits and their interaction with diet as additional predictors. Each of the performance traits was analyzed in a separate model. In the same way we used *ancombc2* to analyze the association of the performance traits with the bias-corrected “absolute” abundances of particular taxa (phyla and genera), again, by adding the performance traits as additional predictors to the same models as used for comparing the abundances. The correlations of the performance traits with relative abundances of the particular phyla and genera were tested in more intuitive way, by fitting linear models (R *lm* function) with the performance traits as the dependent variable, and the microbiome traits as predictors (and the same set of the fixed predictors as used in analyses aimed at testing the effects of experimental factors on the performance traits). In both of the analyses of correlations with abundances of particular taxa, P-values were corrected using False Discovery Rate correction (“BH” option in R function *p.adjust*).

2. Supplementary Results

2.1. The Ci-present microbiome

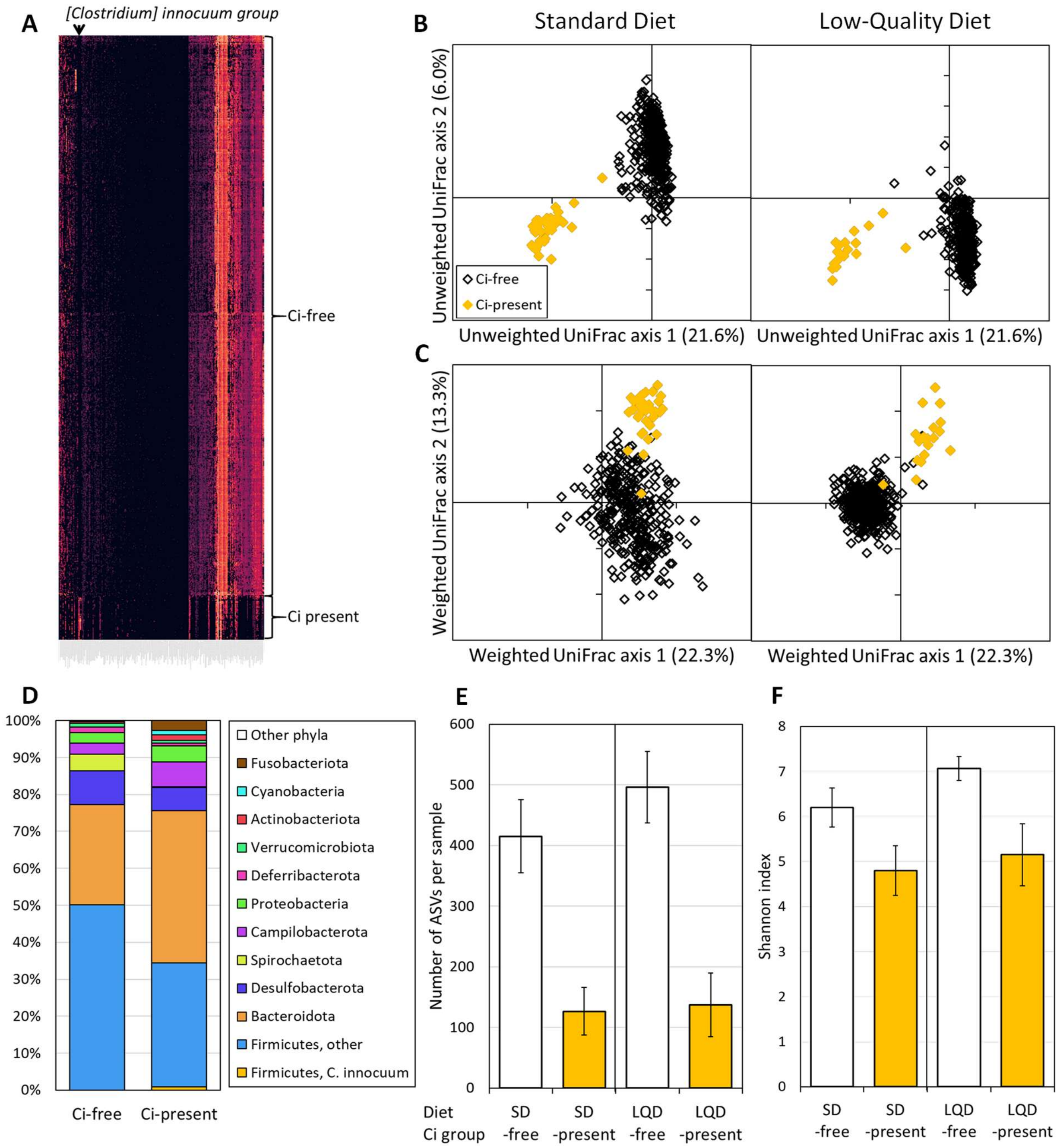

Fig. S2 Caecal microbiome characteristics in bank voles distinguished by presence or absence of bacteria from the [*Clostridium*] *innocuum* group (Ci). **A)** heatmap of abundances of bacterial genera (bacterial genera on horizontal axis, individuals on vertical axis; **B,C)** scores of microbiomes of individual voles on the first two Principal Coordinates Analyses (PCoA) axes based on unweighted **(B)** and weighted **(C)** UniFrac distances (for a better clarity, each displayed on two panels split by diet, but PCoA was performed for all individuals together); **D)** relative abundance of main bacterial phyla ([*Clostridium*] *innocuum* group highlighted within the Firmicutes phylum); and Mean ± SD of **E)** the number of amplicon sequence variants (ASVs) and **F)** Shannon index. The phyla names on the figure are such as in the SILVA 138 database, but according to current nomenclature some have different names: Firmicutes = Bacillota, Desulfobacterota = Thermodesulfobacteriota, Proteobacteria = Pseudomonadota, Actinobacteriota = Actinomycetota, Cyanobacteria = Cyanobacteriota.

Preliminary analyses revealed a group of 55 voles (7.4%) with a strikingly distinct bacterial community membership and structure (Fig. S2A,B), which could be nearly perfectly separated by a single criterion, the presence of bacteria from *[Clostridium] innocuum* group. The microbiome of individuals from this group was less diverse, as shown by a significantly lower number of ASVs (N_{ASV}) and Shannon index (Fig. 2E,F), and lower Shannon diversity and Faith's phylogenetic diversity index (Supplementary Table S4).

In the *Ci*-free voles (690 individuals), the majority of the bacterial community was formed by two phyla: Firmicutes (50.2%) and Bacteroidota (27.1%), whereas in the 55 *Ci*-present voles, the rank of these phyla was inverted (Bacteroidota 41.3%, Firmicutes 34.4%; Table S3, Fig S2D). The 55 *Ci*-present voles were distributed nearly equally across cage types and sexes (chi-square test of independence performed separately for each of the factors, p≥0.27), but *Ci-present* phenotype was more common in the group fed SD than LQD (10% vs. 5%; chi-square p=0.026). However, they were present in only 21 out of 148 families of the voles, and in 8 of these families all individuals belonged to the *Ci*-present category. The association of the *Ci* presence with family was distinctly non-random (chi-square test with p values based on Monte Carlo randomization, p<1E-6). With such a strong family effect and the overall low proportion of *Ci-presence*, individual-level analyses would be ineffective in answering the question whether the Ci-presence is associated with linetype. Instead, an analysis of frequencies at the level of the full-sib families, in which either at least one individual belonged to the *Ci*-present group *vs* those in which all individuals were *Ci*-free, could be applied. The *Ci*-present families appeared less frequently in the H lines (9/70 families, 13%) than in the C lines (12/57 families, 21%), but the difference was not statistically significant (p=0.35).

The *Ci*-present voles had also a significantly lower body mass at the onset of the feeding trial 20.8±1.22g, vs. 22.0.9±1.05; p=0.007), and a 3.1% points lower apparent food digestibility (p<0.001).

Although we operationally used presence of the bacteria from the [*Clostridium*} *innocuum* group to distinguish the *Ci*-present and *Ci*-free categories, we do not claim that the presence of this particular bacteria was the causal factor behind the distinct microbiomes. Although it is tempting to hypothesize that an infection with this specific bacterium has led to the extinction or decreasing abundance of many bacterial taxa, and has created conditions in which only a few other taxa found favorable conditions (e.g., *Fusobacterium*, which was nearly absent in *Ci*-free voles, or an unnamed genus representing Muribaculaceae, whose abundance grossly increased in the *Ci*-present group; Table S3), the direction of the causal effect could be reversed. Moreover, the development of the distinct microbiome may have been initiated by specific physiological conditions in the vole's caecum, whether determined genetically or environmentally, rather than by the invasion of a particular bacterial species. We have observed a similarly distinct microbiome in a comparable proportion of voles in other studies based on our selection experiment (Lipowska et al., 2024; Hämäläinen et al., unpublished). Thus, the specific microbiome did not result from an incidental infection during this experiment.

2.2. The relation between feeding-trial traits and body mass

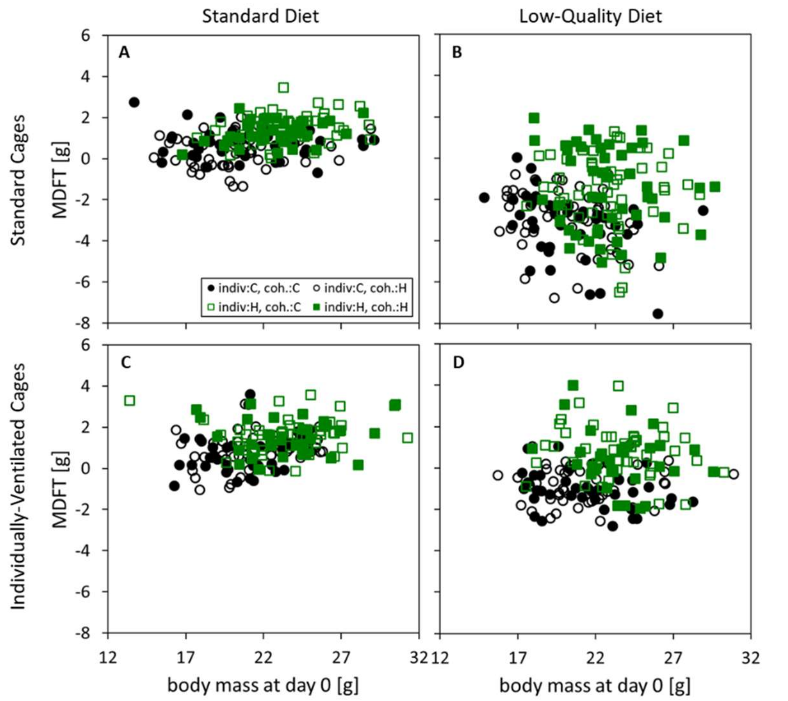

Fig. S3 The relationship between body mass change during the feeding trial (MD_{FT}) and initial body mass, in animals tested in standard cages (top row) or individually-ventilated cages (bottom row) and fed either standard diet (left column) or low-quality diet (right column).

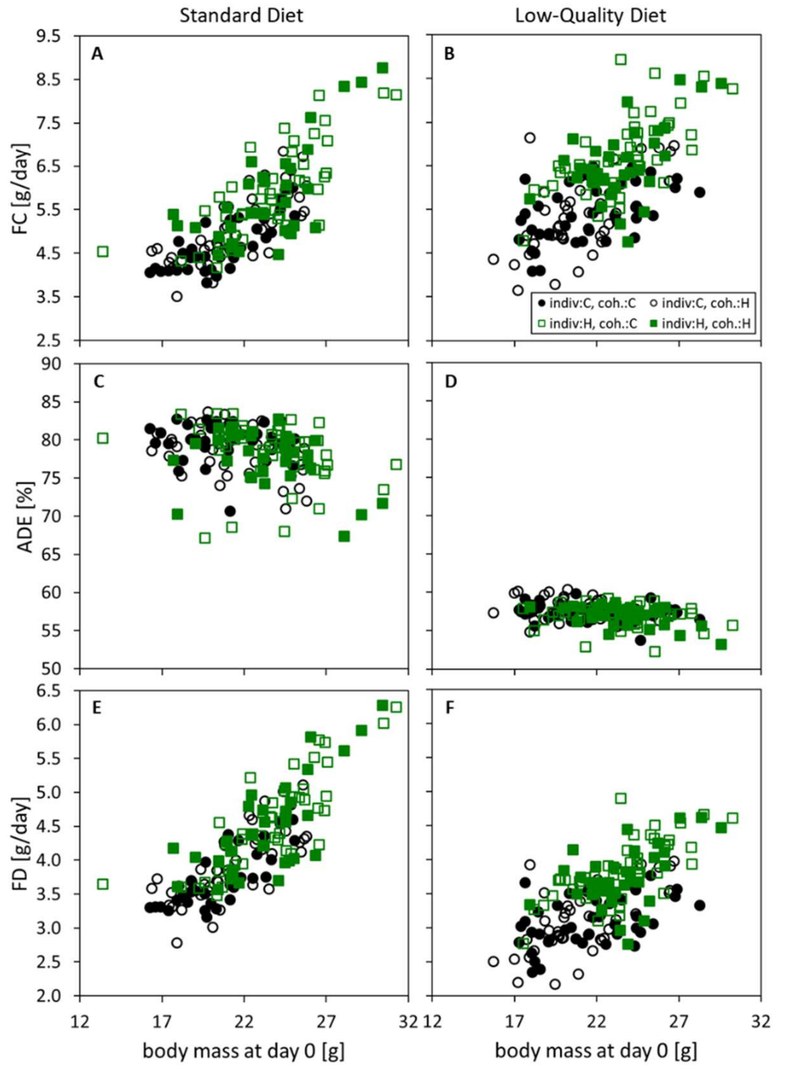

Fig. S4 The relationship between feeding trial performance traits and initial body mass in animals tested in the individually-ventilated cages and fed either standard diet (left column) or low-quality diet (right column). FC - rate of food consumption, ADE – apparent digestive efficiency, FD – rate of food digestion (ADE = 100 × FD/FC).

2.3. Abundances of the bacterial taxa and correlations between the host performance and the abundances of microbial traits

2.3.1. Abundances of the bacterial taxa

We analyzed abundances of 11 phyla and 115 genera with univariate models using two metrics: untransformed relative abundances, and bias-corrected absolute abundances (ANCOM-BC2) (Fig. 4; Table R1; Tables S7, S9-10, S12-13). Diet type affected the relative and/or absolute abundances of all the phyla except Deferribacterota, and most of the genera and ASVs. Both methods showed that eating the LQD increased the abundances of Thermodesulfobacteriota [*SILVA 138* database name: Desulfobacterota] and the Candidate Phyla Radiation group [Patescibacteria], and decreased that of Spirochaetota, Pseudomonadota [Proteobacteria] and Verrucomicrobiota. The relative abundances (but not absolute) of several taxa appeared significantly associated with sex, or differed between cage types.

Irrespective of these confounding factors, the selection significantly affected the relative or absolute abundances of 8 phyla and 61 genera (Fig. 4; Table R1 (below); Excel file Supplementary Tables S7, S9-10, S12-13). Voles from the H lines, irrespective of the linetype of the cohabitant, had higher relative abundances of Pseudomonadota (Proteobacteria; C: 2.6%, H: 3.2%), Deferribacterota (C: 1.3%, H: 1.8%, p=0.001), Thermodesulfobacteriota (Desulfobacterota; C: 8.5 %, H: 9.6%, p=0.001), and Actinomycetota (Actinobacteriota; C: 0.4%, H: 0.5%, p=0.034; Fig. 4A, 5), and for the first two of these phyla the absolute abundance was also significantly increased. On the other hand, H-line voles had lower relative and absolute abundances of Bacteroidota (C: 27.6%, H:26.6%; p=0.034), Campylobacterota (C: 3.2%, H:2.7%, p=0.047), a lower absolute abundance of Verrucomicrobiota (C: 1.1%, H:1.0%; log-fold change = -0.14 ± 0.06. p=0.037), and tended to have a lower relative and absolute abundance of Cyanobacteriota (Cyanobacteria; C: 0.21%, H: 0.17%; p=0.057; log-fold change = -0.12 ± 0.06. p=0.064). The abundance of Bacillota [Firmicutes] did not differ between the H and C lines (p=0.8), but within this phylum the selection affected both the relative and absolute abundances of 8 genera, only the relative abundance in 31 genera, and only absolute abundance in one genus (Fig. 4B, 5, Table R1). For example, in the H lines the abundances of *Acetatifactor, Ruminococcus, Lactobacillus, Ileibacterium,* and *Anaeroplasma* were increased, and those of *Roseburia, Blautia,* and *Fournierella* were decreased. In other phyla, the selection affected both the relative and absolute abundances of 7 genera, only the relative abundance in 12 genera, and only absolute abundance in 2 genera were affected by the selection. The abundances of *Desulfovibrio*, *Mucispirillum*, *Bifidobacterium* and were increased in H lines, whereas those of *Rikinella*, Muribaculaceae, and *Bilophila* were decreased.

The cohabitant selection line type did not significantly affect the relative abundance of any of the analyzed phyla or genera. Analyses of absolute abundance showed an effect of cohabitant for one genus (Veillonellaceae_UCG-001) in analyses of both diets combined. However, the results for this genus are unreliable. The log-fold change value (+0.33) is 50% higher than that of any other genus. The values obtained from the analyses performed separately for the two diet groups are much smaller and in opposite directions (SD: +0.09, LQD: -0.08). It is also the only genus in which the significant effect of the focal individual's selection line on absolute abundance is opposite in the SD and LQD groups. Additionally, this result does not align with the analyses of relative abundances, which consistently showed significantly lower relative abundance in H lines. Therefore, all the results for this genus are suspect. Thus, the results did not provide compelling evidence of the effect of cohabitant linetype on the abundance of any genus.

The abundances of two phyla and many genera depended on interacting factors, especially those related to diet type (Table R1; Tables S7, S9-10, S12-130). For instance, the relative abundance of Proteobacteria (Pseudomonadota) and Actinobacteriota (Actinomycetota) was much higher in H-line voles than in C-line voles in the group fed SD, but not in the group fed LQD. *Lactobacillus* abundance was much higher in animals fed an SD diet. It was also significantly higher in the H lines than in C lines in both diet groups, but the magnitude of the difference differed significantly between the diet groups (mean ± s.d.: C lines, SD: 7.23 ± 3.96%, LQD: 1.24 ± 1.43%; H lines, SD: 9.78 ± 4.22%, LQD: 2.15 ± 2.08%; interaction p = 0.009; Table S10). Similar patterns appeared in many genera. In several cases where a significant interaction occurred, analyses split by diet showed that the effect of selection line type was significant in only one diet group. However, as with *Lactobacillus*, the direction of the significant effects of selection was consistent for both diet groups in all but one genus. The single exception appeared in the analysis of the absolute abundance of Veillonellaceae_UCG-001, but this result is questionable (as explained in the previous paragraph).

2.3.2 Abundances of amplicon sequence variants (ASVs)

We analyzed the relative and absolute abundances of 1344 ASVs that were present in at least 10% of *Ci-free* individuals. These ASVs represent 26% of the total of 5150 ASVs observed in *Ci-free* individuals, but 97.5% of the total number of amplicon sequence reads in these individuals (Tables S8, S11, S14). Diet affected significantly relative abundance of 909 (68%) and absolute abundance of 713 (53%) of these ASVs.

More importantly, the relative abundance of 427 ASVs (47% of all analyzed) was significantly affected by the selection linetype of the focal individual, either in the analyses for combined diets or separate for the diet groups. Among those ASVs, in 356 (26% of all analyzed) the direction of the difference between the H and C lines was the same in the SD and LQD diet groups, which shows consistency of the effect of selection (Tables S8, S11).

In many cases where selection did not affect a genus's abundance, the abundances of several of its ASVs differed in opposite directions between the H and C lines. Furthermore, in most genera in which abundances did not differ significantly between selection line types, abundances of some ASVs differed significantly in opposite directions. These opposite trends were also common within species. These patterns are evident in Table R2 (this file, below), where we showed simplified results for the effect of selection on the relative abundances of genera and ASVs, for a limited number of 250 ASVs that met the following criteria: a) showed a significant effect of selection for at least one diet group or in the analysis for combined diets; b) mean abundances differed between voles from the selected H lines and control C lines consistently in the SD and LQD diet groups; c) had a relative abundance above 0.01% in at least one of the selection × diet subgroups; and d) at least two ASVs meeting the three criteria represented a genus.

Such results suggest that the selection for the capability to cope with the low-quality diet not only affected the overall abundances of many bacterial genera, but also modified the composition of bacterial lineages within the genera and species. However, to analyze possible functional associations of genetic differences between the bacterial strains and the selected trait the analysis would have to be based on metagenomic data, rather than only the sequences of amplicons of the V4 region of the 16S ribosomal RNA gene.

Several ASVs were present nearly exclusively in the selected H lines. The most striking example is an ASV from an uncultured genus of Puniceicoccaceae (ITax = 5600 in Tables S8, S11, S14), which was

present in only 3 voles from the C lines, and all these individuals were cohabited voles from the H lines. On the other hand, it was present in 154 individuals (42%) from the H lines: 82 (41%) cohabited with C-line voles, and 72 (43%) cohabited with another H-line voles. Similar, although less striking examples are ITax 2864 of an uncultured genus of Oscillospiraceae (Firmicutes = Bacillota) or ITax 4908 of a genus of Muribaculaceae (Bacteroidota). Such results suggest the possibility of evolution of new, genetically distinct lineages of these bacterial species. Again, this conjecture cannot be tested more rigorously based on the available data, because this would require information of the ASVs in the base population of the selection experiment.

The pattern revealed in the analyses of absolute abundances was similar, but less ASVs showed significant effect of selection: in 120 ASVs (9%) the effect was significant in at least one diet group or in analysis for combined groups, and in 70 of those the direction of the effect of selection was consistent for both SD and LQD diets (Table S14).

In contrast to the results showing the significant effect of selection linetype of the focal individual on the abundances of many ASVs, the linetype origin of cohabitant did not affect significantly the relative abundance of any ASV (Table S11), and affected the absolute abundance of only 27 ASVs, of which in only 10 the direction of the effect was consistent in animals fed the SD and LQD diet (Table S14).

To summarize, the results of univariate analyses of the abundances of ASVs reinforced the conclusion that the microbial composition of the voles is determined mostly by diet and their selection linetype origin (H *vs* C lines), and is only weakly affected by bacterial transfer from cohabited individual. Such results support the hypothesis that the microbial composition is subject to control mechanisms of the host, and the selection has led to a shift in the controlled composition.

2.3.2 Individual-level correlations between the bacterial characteristics and the host traits

The individual-level phenotypic correlations were analyzed as the partial regression slopes in models that included the same sets of predictors as in the models used for testing the traits of interest. Thus, the correlations reported here are adjusted for the correlations due to the differences between the experimental groups.

Body mass change during the feeding trial (MD_{FT}) in animals kept in standard cages was not correlated with any of the alpha diversity metrics, and in those kept in the IVC metabolic cages the correlations differed between diet groups (significant interactions; Table S15). In animals fed LQD, MD_{FT} was negatively correlated with the Shannon index ($p=0.034$), Shannon diversity ($p=0.041$), and Pielou index ($p=0.003$), whereas in those fed SD it was negatively correlated with the Faith's phylogenetic diversity ($p=0.038$). In those fed the standard diet the trends were opposite, but not significant. The rates of food consumption (FC) and apparent digestion (FD) were not correlated with the alpha-diversity indices, but the apparent digestive efficiency (ADE) increased with N_{ASV} ($p<0.001$) and with Shannon index ($p=0.022$; Fig. S5A,B; Table S15).

Multivariate analyses showed that MD_{FT}, especially in animals fed LQD, was correlated with both of the beta-diversity metrics, the community membership (characterized by the unweighted UniFrac distances) and community structure (weighted UniFrac distances; Table 1). FC and FD were correlated with the community membership, whereas ADE was correlated with both the community membership and structure (Table 1).

Univariate analyses of partial correlations revealed that MD_{FT} and ACE, but not FC and FD, were correlated with abundances of several phyla and genera. As could have been expected, the correlations were often complicated by significant interactions with diet; in such cases, and also when

the number of individuals with non-zero relative abundances in a dietary group was less than 10%, we report results for the SD and LQD groups separately (but estimated with the framework of models including both groups; Table R1 below in this file; Tables S15, S16).

The MD_{FT} of animals kept in standard cages and fed SD was not dependent on the relative or absolute abundance of any phylum ($p \geq 0.8$). However, in animals fed LQD, i.e., under conditions in which MD_{FT} is measured as the selection criterion, MD_{FT} correlated with the relative abundance of seven bacterial phyla (Table R1; Table S15). It increased with the relative abundance of Thermodesulfobacteriota [Desulfobacterota], Spirochaetota. Pseudomonadota [Proteobacteria], Verrucomicrobiota and Actinomycetota [Actinobacteriota], and decreased with abundance of Bacteroidota ($0.001 \leq p \leq 0.046$; Fig. S5 E-H; Table R1, Tables S15, S16). Associations with the absolute abundance had the same directions, but the correlation was significant only in Verrucomicrobiota. Abundance of Bacillota [Firmicutes] was not correlated with MD_{FT}, but abundance of several genera in this phylum was correlated with this trait. In LQD group, MD_{FT} was significantly correlated with relative abundance of 19 genera (e.g., positively: *Roseburia*, *Tyzzerella*, Clostridia_vadinBB60_group, *Alistipes*, *Desulfovibrio*, an uncultured Rickettsiales, unclassified Puniceicoccaceae; negatively GCA-900066575, *Tuzzerella*, *Colidextribacter*, *Peptococcus, Streptococcus*, a Muribaculaceae, and an uncultured Desulfovibrionaceae). The correlations with the absolute abundance had in nearly all cases the same directions as those for the relative abundances, but they were only significant for three genera, in which the relative abundance was also significantly correlated (Tyzzerella, Streptococcus, and an unclassified Puniceicoccaceae). In SD group, MD_{FT} was not correlated with relative abundance of any genus, but it was correlated with the absolute abundances of 3 genera. In *Streptococcus* and an unclassified Pasteurellaceae the correlations were consistent with those for LQD group, and in *Bifidobacterium*, which present practically only in SD-fed voles, the correlation was negative (Table R1, S16). In animals kept in the IVC metabolic cages, MD_{FT} was not significantly correlated with the relative abundances in either of the diet groups, but correlations with the absolute abundance was present for a few genera. In SD-fed voles MD_{FT} decreased with the absolute abundance of *Anaerofilum*, *Rikenella*, *Nitrosomonas*, and *Rothia*. In those fed LQD, it decreased with the abundance of Candidatus_Arthromitus, Veillonellaceae_UCG-001 and *Bauldia*, and increased with abundance of *Ureaplasma*.

The rate of food consumption (FC), the apparent digestive efficiency (ADE), and the rate of effective food digestion (FD) were not significantly correlated with either the relative or absolute abundances of any phyla, except that in voles fed SD the ADE decreased with relative abundance of Bacteroidota ($p<0.001$; Table R1, Tables S15, S16). The correlations of FC and FD with abundances of genera were also scarce, but ADE was correlated with abundances of several genera. Irrespective of whether the correlations were significant or not, the direction of the correlations with ADE was usually opposite to that with FC (which is at least partly a consequence of principally negative correlation between FC and ADE). FC was correlated significantly with the relative abundance of only three genera of Firmicutes. In both dietary groups, FC decreased, while ADE increased with relative abundance of UCG-010, but the gain of increased ADE did not compensate the decreased FC, and FD decreased with increasing abundance of this genus. On the other hand, FC increased with the abundance of an uncultured genus of Lachnospiraceae and a Ruminococcaceae Incertae Sedis, and despite negative correlation with ADE, FD increased with the abundance (although the correlation was significant only in the latter genus). In addition, in voles fed SD, FD significantly increased with relative abundance of *Lactobacillus*, although ADE decreased (Fig. S5 C,D).

In both diet groups, ADE was correlated with the relative or absolute abundance of 7 genera (positively: *Ruminococcus*, UCG-010, an unclassified Christensenellaceae, and unclassified Desulfovibrionales; negatively: Lachnospiraceae_FCS020_group, unclassified Oscillospiraceae, and Ruminococcaceae Incertae Sedis; Table R1; Tables S15, S16). In SD-fed animals, ADE also increased with the abundance o6 5 genera (Lachnospiraceae UCG-001, [Eubacterium]_coprostanoligenes_group, *Ileibacterium*, Rs-E47 termite group, *Trepomema,* and *Enterorhabdus*) and decreased with the abundance of 11 genera (an uncultured and an unclassified Lachnospiraceae, *Lachnoclostridium*, [Eubacterium]_xylanophilum_group, *Acetatifactor*, *Colidextribacter*, *Anaerovorax, Monoglobus*, *Lactobacillus*, *Streptococcus***,** a Muribaculaceae). Only in the LQD group, ADE increased with the abundance of uncultured Christensenellaceae and unclassified Cellvibrionaceae.

2.2.3. Functional analysis of the differences in abundances between the selected and control lines and of the correlations between the abundances and the host performance traits

The abundances of Thermodesulfobacteriota (Desulfobacterota; mainly *Desulfovibrio*), Pseudomonadota (Proteobacteria; uncultured genera of Rickettsiales and Paracaedibacteraceae), and Actinomycetota (Actinobacteriota; mainly *Bifidobacterium*) increased in the H lines, and were positively correlated with the body mass change in the feeding trial. While it is unclear how the Pseudomonadota could be beneficial, the possible beneficial effects of *Bifidobacterium*, which was nearly absent in the C lines but increased to a substantial 0.12% of relative abundance in the H lines, are readily apparent. The bacterium is well known to be involved (directly or through interactions with other bacteria) in degradation of fibrous carbohydrates and the production of short-chain fatty acids (SCFAs; mainly butyric and acetic) and lactate, which become a source of energy, as well as in protection against pathogens, production of vitamin B, antioxidants, and stimulation of the immune system in humans (Rivière et al. 2016), and therefore it is included in probiotic supplements. *Desulfovibrio* has both beneficial and adverse effects in humans (Singh et al. 2023), but it is a potent producer of acetic acid and other SCFA (Hong et al. 2021), and is also involved in hydrogen scavenging (Kohl et al. 2016), which again may increase the efficiency of using the fibrous carbohydrates as a source of energy. The abundance of Bacillota (Firmicutes), the most abundant phylum, did not change systematically in response to selection, but several of its genera that increased abundance in the H lines can be also associated with plausible beneficial roles, not only associated with digestion. For example, *Acetatifactor*, in addition to producing SCFA, is involved in stress response (Liu et al. 2022; Ma et al. 2024), and its increased abundance suggests a lower stress level in voles from the H lines, which can contribute to the improved ability to maintain energy balance under the conditions of suddenly worsened diet. *Ruminococcus* is involved in fiber degradation (Christopherson et al. 2014), and *Lactobacillus*, which was positively correlated with the rate of effective food digestion, a proxy of metabolizable energy intake, is a the dominant genus in the foregut chambers of several herbivorous rodents (Kohl et al. 2014; Shinohara et al. 2016) and is associated with growth promotion in malnourished mice through interactions with hepatic growth hormone signaling (Schwarzer et al. 2016).

On the other hand, the abundance of Bacteroidota, the second most abundant phylum, was decreased in the selected H lines, and at the level of individual variation it was negatively correlated with both the body mass change in the trial with low-quality diet, i.e. with the value of the selected trait, and with the apparent digestive efficiency (this latter correlation concerned the standard rather than the low-quality diet, but, as we have explained above, this is not surprising). A similar pattern was revealed by analyses of abundance of two genera from this phylum (a genus of Muribaculaceae, the most abundant in this phylum, and a less abundant *Rikenella*), and, not so clearly, in analyses of abundance

of two less common phyla: Campylobacterota (mainly *Helicobacter*), and Cyanobacteriota (Cyanobacteria; mainly *Gastranaerophilales*). These results can be taken as suggesting that high abundance of these bacteria compromise the efficiency of digestion, and consequently the energy balance and the growth rate, and therefore the selection favored voles that are able to control the abundance of these bacteria at a lower level. Such an interpretation is strengthened by known functional effects of some of these bacteria. The involvement of *Helicobacter* in compromised gastrointestinal function in humans is widely known, and such effects appear also in rodents (see references in: (Charles River 2011)). Thus, its decreased abundance may be associated with increased digestive performance and generally improved health. The abundance of *Rikinella* is decreased in gut microbiome of obese humans (Palmas et al. 2021), which implies a negative association of this genus abundance with energy balance or predisposition to store energy reserves, or both. A propensity to keep a positive energy imbalance and to accumulate fat are considered adverse in the context of contemporary human health, but these are exactly the characteristics favored in our experimental evolution model. Actually, according to the “thrifty genotype hypothesis”, which attempts to explain evolutionary basis of the human propensity to develop obesity when access to energy-rich food is unlimited and physical activity reduced, the same concerned also our human hunter-gatherer ancestors (Chakravarthy and Booth 2004).

Table R1. Simplified summary of the effects of selection abundances of bacterial phyla and genera, and correlations between these abundances and physiological performance traits: body mass change (MDFT) during the feeding trial and apparent digestive efficiency (ADE). Prev. % - mean prevalence, Log10 RA% - log10(mean relative abundance, %); SC - standard cages, IVC - individually ventilated metabolic cages, SD - standard diet; LQD - low-quality diet. **The effects are shown as:** plus: positive effect (H lines > C lines, or positive correlaton); minus: negative effect (H lines < C lines or negative correlation); ++ or ––: p<0.01; + or –: p<0.05; (+) or (–): significant overall effect for combined diets and the same direction of effects for both diets, but the effect conisidered separately for a diet not significant (0.05<p<0.5). Complete results are presented in Supplementary Tables S5, S9-16

					effect of selection				correlations											
			Prev.	Log10					MD_{FT}, SC				MD_{FT}, IVC				ADE			
			%	RA %	SD		LQD		SD		LQD		SD		LQD		SD		LQD	
					relative (R) or absolute (A) abundace															
ITax	Taxon				R	A	R	A	R	A	R	A	R	A	R	A	R	A	R	A
	**Phylum**																			
1	**Fir**micutes		100	1.7																
2	**Bac**teroidota		100	1.4	(–)	(–)	(–)				––						––			
3	**Des**ulfobacterota		100	1.0	++		(+)				+									
4	**Spi**rochaetota		97	0.7							+									
5	**Cam**pilobacterota		86	0.5	(–)		(–)													
6	**Pro**teobacteria		100	0.5	++	++					+									
7	**Def**erribacterota		94	0.2	++	++	(+)													
8	**Ver**rucomicrobiota		94	0.0		(–)	––				++	++								
9	**Act**inobacteriota		100	-0.3	++						+									
10	**Cya**nobacteria		92	-0.7	–								(–)	–						
11	**Pat**escibacteria		81	-1.4																
	(Phylum abbrev.) **Genus**																			
159	Fir.	Lachnospiraceae_NK4A136_group	100	0.9																
160	Fir.	uncultured f. Lachnospiraceae	100	0.6	(–)		(–)										––			
161	Fir.	unclassified f. Lachnospiraceae	100	0.4													–			
162	Fir.	Roseburia	100	0.4	(–)		(–)				+									
163	Fir.	Lachnospiraceae_UCG-001	99	0.2													++			
164	Fir.	Blautia	99	-0.2	–		(–)													
165	Fir.	Lachnoclostridium	100	-0.3													––			
166	Fir.	A2	93	-0.3																
167	Fir.	[Eubacterium]_xylanophilum_group	97	-0.3													–			
168	Fir.	Lachnospiraceae_UCG-006	99	-0.4	(–)		(–)													
169	Fir.	Tyzzerella	97	-0.4							++	+								
170	Fir.	ASF356	87	-0.5																
171	Fir.	Coprococcus	22	-0.5																
172	Fir.	Acetatifactor	80	-0.6		(+)	(+)										––			
173	Fir.	GCA-900066575	96	-0.7							––									
174	Fir.	Lachnospiraceae_NK4B4_group	83	-0.8																
175	Fir.	Lachnospira	62	-1.0																
176	Fir.	Tuzzerella	92	-1.1		(–)					––									
177	Fir.	Lachnospiraceae_FCS020_group	89	-1.1													––		–	
178	Fir.	Acetitomaculum	73	-1.3	+															
179	Fir.	Oribacterium	65	-1.4			+													
180	Fir.	Lachnospiraceae_AC2044_group	70	-1.7																
181	Fir.	[Ruminococcus]_gauvreauii_group	22	-1.9	(–)		(–)													
182	Fir.	[Ruminococcus]_torques_group	32	-2.0	(–)		–													
184	Fir.	Eisenbergiella	49	-2.0	(+)		(+)													

			Prev.	Log$_{10}$	effect of selection				correlations															
									MD$_{FT}$, SC				MD$_{FT}$, IVC				ADE							
			%	RA %	SD		LQD		SD		LQD		SD		LQD		SD		LQD					
					relative (R) or absolute (A) abundace																			
ITax	Taxon				R	A	R	A	R	A	R	A	R	A	R	A	R	A	R	A				
186	Fir.	Lachnospiraceae_UCG-010	21	-2.6	(–)		(–)																	
187	Fir.	[Eubacterium]_fissicatena_group	22	-2.6	(+)		+																	
196	Fir.	uncultured f. Oscillospiraceae	100	0.6	(–)		(–)				––													
197	Fir.	Colidextribacter	100	-0.2							––						––							
198	Fir.	Oscillibacter	100	-0.2	(+)		(+)																	
199	Fir.	UCG-005	97	-0.5							+													
200	Fir.	UCG-003	100	-0.7	––	–	––	–			–													
201	Fir.	NK4A214_group	98	-0.7																				
202	Fir.	unclassified f. Oscillospiraceae	78	-1.5			+										––		–					
203	Fir.	UCG-002	34	-1.5	(–)		–																	
204	Fir.	UCG-007	17	-2.6																				
205	Fir.	Flavonifractor	21	-2.6																				
207	Fir.	uncultured f. Ruminococcaceae	100	0.4			+																	
208	Fir.	Ruminococcus	77	-0.1	(+)		+										+		+					
209	Fir.	Incertae_Sedis	100	-0.2													––		(–)					
210	Fir.	[Eubacterium]_siraeum_group	91	-0.2	+		(+)																	
211	Fir.	UBA1819	99	-0.4																				
212	Fir.	Fournierella	81	-0.5	––	––	(–)																	
213	Fir.	Ruminococcaceae	71	-0.9																				
214	Fir.	unclassified f. Ruminococcaceae	86	-1.4	(+)		++	++																
215	Fir.	Paludicola	45	-2.2																				
216	Fir.	Anaerofilum	29	-2.3			(–)							––										
217	Fir.	Anaerotruncus	34	-2.4																				
219	Fir.	Butyricicoccus	94	-0.9	–																			
220	Fir.	UCG-009	96	-0.9																				
221	Fir.	[Eubacterium]_coprostanoligenes_group	69	-1.2	––		(–)										(+)							
222	Fir.	UCG-010	66	-1.5													++		+					
224	Fir.	unclassified o. Oscillospirales	19	-2.7	+		+																	
226	Fir.	uncultured f. Christensenellaceae	83	0.4																++				
227	Fir.	unclassified f. Christensenellaceae	85	0.4													++		+	++				
228	Fir.	Christensenella	36	-2.4	(+)		++																	
230	Fir.	Clostridia_vadinBB60_group	100	0.4							+													
231	Fir.	uncultured f. Peptococcaceae	94	-0.3																				
232	Fir.	Peptococcus	95	-1.0	–						––													
233	Fir.	Family_XIII_UCG-001	87	-1.1																				
234	Fir.	[Eubacterium]_brachy_group	96	-1.2	+																			
235	Fir.	Family_XIII_AD3011_group	78	-1.4	(+)		++				–													
236	Fir.	unclassified f. Anaerovoracaceae	24	-2.3			+																	
237	Fir.	Anaerovorax	23	-2.6													––							
240	Fir.	Monoglobus	33	-0.7	(+)		+										–							
241	Fir.	unclassified c. Clostridia	87	-1.3																				
242	Fir.	Clostridia_UCG-014	82	-1.3																				
243	Fir.	Candidatus_Arthromitus	15	-1.3												––				++				
245	Fir.	Anaerofustis	12	-3.0																				
247	Fir.	Lactobacillus	95	0.7	++		++										––							

ITax		Taxon	Prev. %	Log_{10} RA %	effect of selection SD R	SD A	LQD R	LQD A	correlations MD_{FT}, SC SD R	SD A	LQD R	LQD A	MD_{FT}, IVC SD R	SD A	LQD R	LQD A	ADE SD R	SD A	LQD R	LQD A
					relative (R) or absolute (A) abundace															
248	Fir.	Streptococcus	56	-1.9						–	––	–					(–)			
254	Fir.	Ileibacterium	18	-0.2	++	++											++	++		
255	Fir.	unclassified f. Erysipelotrichaceae	10	-0.2	––	––														
256	Fir.	uncultured f. Erysipelotrichaceae	27	-0.8	––															
263	Fir.	Anaeroplasma	50	-0.3	++	++	++													
264	Fir.	RF39	86	-1.1																
265	Fir.	Ureaplasma	13	-2.0												++				
274	Fir.	Veillonellaceae_UCG-001	18	-0.9	––	++	––	––								––				
276	Fir.	Syntrophomonas	31	-2.1			+													
278	Bac.	Muribaculaceae	100	1.2	––						––						––			
279	Bac.	Alistipes	100	0.5	+						+									
280	Bac.	Rikenella	69	-0.6			–							––						
281	Bac.	Rs-E47_termite_group	76	0.5	++												++			
282	Bac.	Odoribacter	97	0.2	++															
283	Bac.	unclassified o. Bacteroidales	67	0.2																
286	Des.	Desulfovibrio	100	0.9	+		(+)				+									
287	Des.	Bilophila	98	-0.5	(–)		–													
288	Des.	unclassified f. Desulfovibrionaceae	28	-1.0																
289	Des.	uncultured f. Desulfovibrionaceae	77	-1.5	––		–				––									
290	Des.	unclassified o. Desulfovibrionales	92	-0.3	++	+	(+)										+			+
292	Spi.	Termite_Treponema_cluster	69	0.6																
293	Spi.	Treponema	44	-0.8		–		–									++	++		
295	Spi.	Brachyspira	82	-1.6																
296	Cam.	Helicobacter	86	0.5		–														
297	Pro.	uncultured o. Rickettsiales	100	0.3	++	+					++									
298	Pro.	uncultured f. Paracaedibacteraceae	90	-0.2	++	++														
299	Pro.	Bauldia	17	-1.6												––				
300	Pro.	uncultured o. Rhodospirillales	36	-1.9																
301	Pro.	unclassified f. Cellvibrionaceae	41	-0.7																++
302	Pro.	Oxalobacter	91	-1.1			–													
303	Pro.	Nitrosomonas	84	-1.6										–						
307	Pro.	unclassified f. Pasteurellaceae	23	-2.4						––	–									
315	Def.	Mucispirillum	94	0.2	++	++	(+)													
316	Ver.	unclassified f. Puniceicoccaceae	92	0.0	–	–	––				++	++								
317	Ver.	uncultured f. Puniceicoccaceae	21	-0.9	++	++	++													
319	Act.	Enterorhabdus	100	-0.5													++			
320	Act.	unclassified f. Eggerthellaceae	60	-1.6																
321	Act.	Adlercreutzia	68	-1.7			–													
323	Act.	uncultured f. Coriobacteriales_Incertae_Sec	32	-1.5																
324	Act.	Bifidobacterium	12	-1.0	++	++				––										
325	Act.	Corynebacterium	59	-1.8	++		(+)													
326	Act.	Brachybacterium	18	-2.8																
327	Act.	Rothia	11	-3.0																
330	Cya.	Gastranaerophilales	92	-0.7	–									–						
331	Pat.	Candidatus_Saccharimonas	81	-1.4																

Table R2. Simplified summary information about prevalence and mean abundance of a selected bacterial amplicon sequence variants (ASVs). The results are shown for ASVs that met the following criteria: a) showed a significant effect of selection for at least one diet group or in the analysis for combined diets; b) mean abundances differed between voles from the selected H lines and control C lines consistently for both the standard (SD) and low-quality diet (LQD) groups; c) had a relative abundance above 0.01% in at least one of the selection × diet subgroups; and d) at least two ASVs meeting these criteria represented a genus. **ITax:** index numbers indentifying a given ASV, genus and species in the main database and other tables; **Genus data:** mean prevalence (Prev., %), mean realtive abundance (RA, %) and significance of the difference between the H and C lines, tested in SD annd LQD diet groups. **The effects in genus are shown as:** plus: positive effect (H lines > C lines); minus: negative effect (H lines < C lines); ++ or ––: p<0.01; + or –: p<0.05; (+) or (–): significant overall effect for combined diets and the same direction of effects for both diets, but the effect conisidered separatelly for a diet not significant (0.05<p<0.5); ns+ or ns-: differences not statistically significant; nd: no difference in abundance (resolution 0.01%). **ASV data:** prevalence (% of individuals with a given ASV present) and mean relative abundance (% of total reads in a given individual) in the selection × diet subgroups. The significance of the effect of selection is coded with the same scheme as for the genus data (separatelly for the SD and LQD groups): green: H > C, pink: H < C ; strong green or pink: p < 0.01; dark green or pink: p<0.05; pale green or pink: significant overall effect for combined diets and the same direction of effects for both diets, but the effect conisidered separatelly for a diet not significant (0.05<p<0.5); very delicate green or pink: differences not statistically significant. Complete results are presented in Supplementary Tables S5,6,10-11.

Genus data						Sp.	ASV	ASV prevalence %				ASV mean abnundance %			
	Taxon	Prev	RA	diff. H-C				C		H		C		H	
ITax	Phylum; **Genus**	%	%	SD	LQD	ITax	ITax	SD	LQD	SD	LQD	SD	LQD	SD	LQD
159	Firmicutes; Lachnospiraceae_NK4A136	100	8.29	ns-	ns-	334	587	1	2	5	19	0.000	0.013	0.001	0.089
159						335	600	26	57	49	75	0.050	0.980	0.100	1.510
159						335	602	62	70	51	61	0.350	0.920	0.220	0.690
159						335	607	96	66	96	59	0.390	0.160	0.340	0.080
159						335	608	93	73	93	70	0.490	0.050	0.380	0.040
159						335	611	7	10	23	33	0.030	0.100	0.140	0.410
159						335	616	25	20	41	26	0.040	0.065	0.095	0.104
159						335	619	73	20	67	13	0.161	0.015	0.115	0.004
159						335	620	28	58	40	66	0.017	0.091	0.020	0.147
159						335	621	11	17	24	34	0.020	0.080	0.044	0.127
159						335	625	22	24	40	46	0.039	0.038	0.067	0.092
159						335	637	35	23	34	28	0.040	0.055	0.017	0.025
159						335	656	36	53	31	37	0.012	0.028	0.008	0.015
159						335	663	75	28	58	12	0.025	0.004	0.022	0.002
159						335	666	13	24	25	46	0.003	0.014	0.006	0.025
159						335	678	5	3	11	17	0.003	0.002	0.007	0.022
159						335	679	48	24	34	14	0.017	0.007	0.009	0.003
159						335	680	64	39	54	26	0.013	0.008	0.010	0.003
159						335	681	14	20	6	10	0.011	0.015	0.002	0.004
159						335	700	7	16	21	37	0.001	0.005	0.003	0.014
159						335	712	30	13	28	9	0.013	0.002	0.004	0.001
160	Firmicutes; uncultured Lachnospiraceae	100	4.43	(–)	(–)	336	1043	11	54	7	41	0.002	0.083	0.001	0.037
160						337	1069	91	100	91	98	0.040	0.200	0.030	0.140
160						337	1074	75	64	83	60	0.118	0.054	0.101	0.024
160						337	1075	80	80	64	59	0.067	0.123	0.034	0.065
160						337	1087	4	23	3	14	0.068	0.057	0.020	0.018
160						337	1090	65	38	56	24	0.090	0.013	0.043	0.008
160						337	1091	60	64	52	38	0.050	0.055	0.020	0.026
160						337	1092	43	5	35	2	0.102	0.006	0.044	0.001
160						337	1093	65	72	62	68	0.043	0.044	0.035	0.027
160						337	1102	11	25	25	45	0.009	0.016	0.015	0.065
160						337	1106	52	50	54	41	0.039	0.019	0.028	0.007
160						337	1111	4	32	7	44	0.001	0.025	0.002	0.051

Genus data						Sp.	ASV	ASV prevalence %				ASV mean abnundance %			
	**Taxon**	Prev	RA	diff. H-C				C		H		C		H	
ITax	Phylum; **Genus**	%	%	SD	LQD	ITax	ITax	SD	LQD	SD	LQD	SD	LQD	SD	LQD
160						337	1113	52	68	51	64	0.012	0.034	0.009	0.021
160						337	1116	9	10	22	23	0.003	0.005	0.016	0.046
160						337	1119	28	64	40	73	0.003	0.027	0.006	0.032
160						337	1120	64	34	46	13	0.017	0.026	0.014	0.010
160						337	1126	51	17	38	8	0.030	0.002	0.027	0.001
160						337	1135	45	80	36	65	0.012	0.018	0.005	0.014
160						337	1137	6	9	20	27	0.002	0.010	0.008	0.027
160						337	1139	50	57	36	43	0.008	0.019	0.006	0.012
160						337	1143	32	25	32	16	0.021	0.008	0.013	0.003
160						337	1150	55	72	40	63	0.007	0.014	0.004	0.011
160						337	1152	46	54	30	38	0.007	0.017	0.004	0.008
160						337	1154	8	30	19	53	0.002	0.007	0.004	0.021
160						337	1163	8	43	2	19	0.002	0.021	0.000	0.005
160						337	1164	4	54	5	41	0.001	0.018	0.000	0.009
161	Firmicutes; unclassified Lachnospiraceae			ns+	ns-	339	1659	73	77	61	57	0.090	0.330	0.080	0.200
161						339	1660	38	24	57	41	0.270	0.020	0.330	0.060
161						339	1662	86	77	75	82	0.067	0.125	0.049	0.109
161						339	1664	2	9	8	27	0.001	0.046	0.023	0.163
161		100	2.35			339	1665	17	30	35	51	0.015	0.024	0.071	0.099
161						339	1679	4	55	9	62	0.000	0.041	0.001	0.060
161						339	1683	5	12	12	29	0.003	0.015	0.008	0.046
161						339	1685	50	24	40	13	0.040	0.006	0.024	0.002
161						339	1703	27	54	16	47	0.003	0.014	0.002	0.010
161						339	1705	41	39	29	22	0.016	0.006	0.004	0.002
162	Firmicutes; Roseburia	100	2.32	(–)	(–)	340	2021	55	23	47	11	0.023	0.006	0.018	0.002
162						341	2035	53	53	57	47	0.089	0.095	0.069	0.040
162						341	2036	21	27	33	35	0.023	0.066	0.048	0.132
162						341	2039	21	73	24	78	0.005	0.138	0.003	0.086
162						341	2040	46	47	18	21	0.047	0.125	0.007	0.037
162						341	2041	68	72	61	68	0.053	0.068	0.032	0.031
162						341	2043	43	53	33	55	0.036	0.064	0.020	0.049
162						341	2044	53	40	39	22	0.079	0.030	0.047	0.012
162						341	2052	14	36	22	56	0.003	0.034	0.006	0.053
162						341	2054	2	9	14	19	0.002	0.013	0.007	0.061
162						341	2057	17	23	27	36	0.007	0.021	0.012	0.042
162						341	2060	10	18	30	46	0.007	0.010	0.029	0.034
162						341	2062	23	31	40	58	0.009	0.019	0.013	0.035
162						341	2073	24	14	4	3	0.030	0.004	0.003	0.000
162						341	2083	58	18	39	11	0.013	0.002	0.008	0.001
162						341	2086	11	23	4	14	0.004	0.011	0.002	0.004
163	Firmicutes; Lachnospiraceae_UCG-001	99	1.55	ns-	ns-	342	2235	57	36	53	36	0.176	0.056	0.104	0.034
163						342	2241	10	10	14	24	0.011	0.026	0.015	0.075
163						342	2253	15	15	11	11	0.010	0.006	0.004	0.003
164	Firmicutes; Blautia	99	0.69	–	(–)	345	2310	86	77	85	59	0.550	0.070	0.350	0.040
164						345	2315	4	8	20	33	0.002	0.009	0.013	0.027
165	Firmicutes; Lachnoclostridium	100	0.53	ns-	ns-	347	2402	92	75	89	58	0.065	0.049	0.059	0.032
165						347	2403	71	69	67	58	0.041	0.056	0.029	0.032
165						347	2405	48	54	38	35	0.015	0.041	0.011	0.018
165						348	2455	28	18	17	8	0.017	0.013	0.006	0.004

Genus data						Sp.	ASV	ASV prevalence %				ASV mean abnundance %			
	Taxon	Prev	RA	diff. H-C				C		H		C		H	
ITax	Phylum; **Genus**	%	%	SD	LQD	ITax	ITax	SD	LQD	SD	LQD	SD	LQD	SD	LQD
166	Firmicutes; A2	93	0.50	ns-	ns-	350	2460	18	35	11	17	0.030	0.250	0.010	0.110
166						350	2463	16	18	15	16	0.011	0.016	0.005	0.005
168	Firmicutes;	99	0.41	(−)	(−)	352	2496	61	66	59	44	0.043	0.044	0.026	0.021
168	Lachnospiraceae_UCG-006					352	2497	26	46	37	64	0.010	0.032	0.016	0.051
168						352	2499	17	35	11	25	0.009	0.058	0.003	0.015
168						352	2502	36	40	21	26	0.010	0.022	0.005	0.013
173	Firmicutes; GCA-900066575	96	0.20	ns-	nd	361	2608	42	73	47	73	0.014	0.071	0.012	0.049
173						361	2609	25	86	40	89	0.004	0.049	0.006	0.075
196	Firmicutes; uncultured	100	3.63	(−)	(−)	393	2783	90	92	93	95	0.160	0.180	0.150	0.130
196	Oscillospiraceae					393	2784	82	66	84	42	0.260	0.070	0.160	0.030
196						393	2787	83	26	83	12	0.240	0.020	0.170	0.010
196						393	2793	30	31	31	29	0.043	0.083	0.021	0.036
196						393	2794	24	51	43	78	0.013	0.043	0.019	0.087
196						393	2795	48	84	61	93	0.012	0.058	0.015	0.076
196						393	2796	72	40	66	23	0.079	0.023	0.052	0.010
196						393	2800	73	67	66	56	0.028	0.040	0.021	0.026
196						393	2802	82	79	81	82	0.030	0.028	0.019	0.023
196						393	2803	29	38	19	24	0.023	0.045	0.010	0.016
196						393	2804	63	77	65	70	0.017	0.035	0.013	0.026
196						393	2810	44	33	40	27	0.022	0.019	0.013	0.013
196						393	2816	14	53	31	77	0.002	0.018	0.004	0.031
196						393	2820	20	29	12	12	0.006	0.031	0.003	0.008
196						393	2821	43	54	25	31	0.011	0.021	0.005	0.011
196						393	2822	49	36	41	26	0.012	0.015	0.009	0.009
196						393	2828	32	58	27	44	0.004	0.014	0.003	0.008
196						393	2829	23	40	14	14	0.007	0.013	0.005	0.003
196						393	2834	11	21	17	34	0.001	0.005	0.002	0.012
196						393	2835	15	47	2	21	0.001	0.015	0.000	0.004
198	Firmicutes; Oscillibacter	100	0.56	(+)	(+)	396	3210	59	34	61	45	0.074	0.052	0.126	0.127
198						396	3213	47	75	37	61	0.067	0.075	0.029	0.055
198						396	3215	16	6	30	14	0.028	0.006	0.073	0.007
198						396	3216	17	21	37	42	0.009	0.017	0.032	0.037
201	Firmicutes; NK4A214_group	98	0.18	ns-	ns+	402	3313	89	97	88	94	0.041	0.134	0.032	0.113
201						402	3314	49	64	64	72	0.024	0.076	0.025	0.117
207	Firmicutes; uncultured	100	2.32	ns+	+	408	3379	95	98	93	99	0.120	1.300	0.180	1.710
207	Ruminococcaceae					409	3406	45	70	59	83	0.008	0.112	0.015	0.181
207						409	3408	26	83	30	93	0.004	0.070	0.006	0.089
207						409	3420	50	77	30	34	0.009	0.025	0.005	0.007
207						409	3421	46	25	27	6	0.020	0.010	0.008	0.001
207						409	3422	49	43	45	29	0.011	0.010	0.009	0.004
208	Firmicutes; Ruminococcus	77	0.84	(+)	+	411	3592	35	39	47	55	0.210	0.460	0.360	0.810
208						411	3596	12	2	26	9	0.019	0.001	0.053	0.012
209	Firmicutes; Incertae Sedis	100	0.64	nd	ns+	413	3607	71	68	63	51	0.084	0.078	0.068	0.033
209	Ruminococcaceae					413	3609	40	28	57	44	0.022	0.040	0.051	0.071
209						413	3611	40	42	59	70	0.019	0.033	0.035	0.067
209						413	3612	89	77	81	63	0.046	0.032	0.038	0.021
209						413	3613	6	24	19	48	0.006	0.024	0.011	0.067
209						413	3615	71	68	67	45	0.031	0.025	0.025	0.014
209						413	3616	80	76	67	57	0.028	0.030	0.020	0.016
209						413	3617	46	50	28	21	0.016	0.045	0.006	0.015
209						413	3618	40	62	49	47	0.017	0.028	0.016	0.014
209						413	3625	29	46	15	28	0.005	0.012	0.003	0.006
209						413	3626	10	33	14	52	0.001	0.007	0.002	0.015
209						413	3627	1	3	11	24	0.000	0.001	0.005	0.013
209						413	3630	12	50	10	31	0.001	0.012	0.001	0.004
211	Firmicutes; UBA1819	99	0.43	nd	ns+	416	3733	15	74	38	78	0.009	0.069	0.024	0.091
211						416	3734	76	50	73	37	0.075	0.014	0.046	0.008

Genus data						Sp.	ASV	ASV prevalence %				ASV mean abnundance %			
	Taxon	Prev	RA	diff. H-C				C		H		C		H	
ITax	Phylum; **Genus**	%	%	SD	LQD	ITax	ITax	SD	LQD	SD	LQD	SD	LQD	SD	LQD
212	Firmicutes; Fournierella	81	0.28	––	(–)	418	3767	46	66	58	83	0.012	0.041	0.014	0.068
212						419	3775	52	38	27	24	0.340	0.180	0.160	0.100
220	Firmicutes; UCG-009	96	0.12	ns-	nd	429	3873	48	57	66	63	0.010	0.010	0.013	0.014
220						429	3874	6	26	14	41	0.001	0.005	0.002	0.011
230	Firmicutes; Clostridia_vadinBB60	100	2.48	ns+	ns-	441	3990	76	92	76	83	0.140	0.280	0.120	0.210
230						441	3991	65	55	59	63	0.220	0.190	0.130	0.130
230						441	3994	76	84	58	57	0.056	0.156	0.047	0.068
230						441	3998	61	59	55	46	0.048	0.053	0.039	0.037
230						441	4004	80	50	77	33	0.061	0.019	0.043	0.011
230						441	4008	45	46	34	36	0.021	0.056	0.014	0.032
230						441	4017	4	25	9	46	0.001	0.029	0.002	0.060
230						441	4019	2	3	20	22	0.002	0.011	0.033	0.044
230						441	4021	1	5	9	31	0.000	0.007	0.004	0.074
230						441	4022	7	14	24	38	0.006	0.016	0.019	0.035
230						441	4026	14	46	25	73	0.002	0.026	0.005	0.037
230						441	4029	13	15	24	27	0.004	0.013	0.014	0.028
230						441	4031	7	31	22	44	0.002	0.022	0.005	0.025
230						441	4032	45	31	48	15	0.027	0.007	0.012	0.003
230						441	4038	12	24	28	46	0.002	0.006	0.005	0.020
230						441	4040	1	1	8	23	0.000	0.001	0.005	0.027
230						441	4043	16	47	13	41	0.006	0.015	0.002	0.007
230						442	4260	24	17	33	24	0.012	0.008	0.022	0.032
230						442	4262	1	10	11	27	0.001	0.015	0.005	0.032
231	Firmicutes; uncultured Peptococcaceae	94	0.47	ns-	ns+	444	4288	90	77	95	75	0.790	0.100	0.690	0.070
231						444	4289	3	13	28	42	0.013	0.019	0.031	0.056
232	Firmicutes; Peptococcus	95	0.11	–	nd	445	4306	60	68	75	67	0.013	0.015	0.018	0.021
232						445	4308	64	53	18	18	0.026	0.019	0.003	0.003
235	Firmicutes; Family_XIII_AD3011	78	0.04	(+)	++	450	4359	30	59	49	77	0.005	0.026	0.011	0.048
235						450	4360	27	30	10	13	0.006	0.013	0.002	0.006
235						450	4361	9	32	17	39	0.001	0.007	0.002	0.010
247	Firmicutes; Lactobacillus	95	5.03	++	++	465	4527	11	2	26	11	0.025	0.004	0.099	0.030
247						467	4530	83	38	86	58	1.170	0.110	1.410	0.240
247						467	4535	33	0	53	2	0.009	0.000	0.021	0.000
247						468	4561	96	49	100	72	1.150	0.150	1.590	0.240
247						468	4562	74	17	83	29	0.680	0.030	0.800	0.110
254						480	4615	20	2	41	7	0.400	0.000	1.460	0.020
254						480	4616	14	1	35	3	0.074	0.000	0.260	0.002
254						480	4617	14	2	33	4	0.056	0.000	0.217	0.002
263	Firmicutes; Anaeroplasma	50	0.48	++	++	491	4650	11	25	28	53	0.030	0.390	0.110	0.790
263						491	4651	15	46	30	67	0.010	0.220	0.020	0.300
278	Bacteroidota; Muribaculaceae	100	16.6	––	ns+	510	4817	98	99	100	99	1.500	1.000	2.000	1.100
278						510	4822	93	79	86	82	0.780	0.580	0.370	0.440
278						510	4823	99	91	99	97	0.420	0.420	0.490	0.510
278						510	4826	98	98	99	99	0.210	0.270	0.280	0.390
278						510	4828	96	95	95	99	0.110	0.270	0.150	0.390
278						510	4834	70	58	49	43	0.170	0.240	0.090	0.170
278						510	4837	30	39	27	32	0.160	0.250	0.090	0.140
278						510	4843	95	39	93	37	0.220	0.070	0.140	0.040
278						510	4845	58	70	76	76	0.020	0.160	0.040	0.180
278						510	4848	85	86	87	92	0.047	0.091	0.066	0.147
278						510	4849	52	7	67	13	0.110	0.012	0.202	0.023
278						510	4850	38	50	29	48	0.074	0.139	0.026	0.087
278						510	4853	54	43	45	47	0.056	0.116	0.030	0.096
278						510	4854	3	11	20	33	0.003	0.058	0.037	0.193
278						510	4856	86	88	90	94	0.040	0.074	0.067	0.103
278						510	4858	63	49	72	55	0.048	0.067	0.083	0.082
278						510	4860	83	79	75	81	0.043	0.079	0.046	0.102

Genus data						Sp.	ASV	ASV prevalence %				ASV mean abnundance %			
	Taxon	Prev	RA	diff. H-C				C		H		C		H	
ITax	Phylum; **Genus**	%	%	SD	LQD	ITax	ITax	SD	LQD	SD	LQD	SD	LQD	SD	LQD
278						510	4868	61	60	56	56	0.079	0.050	0.034	0.024
278						510	4869	79	41	83	68	0.064	0.013	0.077	0.030
278						510	4875	64	23	49	16	0.069	0.023	0.039	0.016
278						510	4876	81	14	81	16	0.047	0.006	0.071	0.008
278						510	4877	75	82	83	92	0.017	0.035	0.020	0.053
278						510	4879	39	54	49	72	0.010	0.037	0.019	0.049
278						510	4880	53	45	54	67	0.019	0.022	0.021	0.052
278						510	4881	55	8	60	14	0.035	0.002	0.066	0.006
278						510	4887	38	21	57	44	0.014	0.009	0.030	0.026
278						510	4888	6	12	8	21	0.002	0.023	0.004	0.045
278						510	4892	54	64	52	74	0.009	0.021	0.012	0.029
278						510	4894	19	25	23	25	0.004	0.017	0.012	0.027
279	Bacteroidota; Alistipes	100	3.53	+	ns-	512	5225	97	96	99	100	0.800	1.800	1.100	1.900
279						512	5227	83	86	90	93	0.460	0.630	0.370	0.480
279						512	5230	49	44	67	68	0.110	0.110	0.160	0.170
279						512	5231	21	25	12	14	0.031	0.057	0.016	0.015
282	Bacteroidota; Odoribacter	97	1.77	++	ns-	517	5279	80	88	94	97	0.200	0.600	0.290	0.630
282						517	5281	1	4	20	27	0.003	0.019	0.061	0.262
282						517	5282	1	4	16	25	0.000	0.002	0.008	0.031
286	Desulfobacterota; Desulfovibrio	100	8.07	+	(+)	522	5300	91	95	90	96	0.220	0.410	0.340	0.550
286						522	5301	15	29	26	38	0.010	0.023	0.038	0.081
286						522	5302	29	43	37	49	0.011	0.039	0.028	0.073
286						523	5323	60	72	76	85	0.110	0.290	0.470	0.650
286						523	5326	57	62	66	73	0.150	0.180	0.270	0.300
286						523	5327	96	99	100	99	0.080	0.310	0.090	0.360
286						523	5329	31	42	67	73	0.020	0.060	0.100	0.260
286						523	5336	74	82	87	95	0.024	0.074	0.048	0.138
286						523	5346	51	87	62	91	0.006	0.022	0.008	0.032
286						523	5350	13	29	28	51	0.002	0.009	0.015	0.021
286						523	5352	2	2	7	18	0.000	0.001	0.011	0.021
286						524	5411	76	80	72	79	0.890	0.980	0.520	0.540
286						524	5413	74	83	79	89	0.130	0.360	0.160	0.400
286						524	5414	45	76	65	85	0.040	0.170	0.060	0.240
286						524	5415	55	72	38	44	0.060	0.240	0.050	0.130
286						524	5416	51	64	68	81	0.048	0.108	0.077	0.159
286						524	5417	30	31	38	47	0.059	0.062	0.087	0.124
286						524	5418	53	50	54	59	0.046	0.063	0.064	0.114
286						524	5419	33	53	44	67	0.025	0.062	0.064	0.131
286						524	5422	22	31	28	41	0.024	0.035	0.067	0.094
286						524	5424	44	91	74	95	0.009	0.054	0.017	0.082
286						524	5425	5	17	21	36	0.002	0.035	0.019	0.086
286						524	5427	26	38	15	11	0.029	0.080	0.010	0.016
286						524	5432	13	64	24	72	0.001	0.018	0.003	0.026
286						524	5437	7	16	2	10	0.002	0.010	0.000	0.002
297	Proteobacteria; uncultured	100	1.87	++	ns+	536	5510	31	40	44	63	0.080	0.300	0.110	0.620
297	Rickettsiales					536	5511	32	46	12	16	0.060	0.370	0.040	0.170
298	Proteobacteria; uncult.	90	0.64	++	ns+	538	5527	49	49	86	81	0.410	0.240	0.910	0.260
298	Paracaedibacterales					540	5529	69	61	76	67	0.073	0.045	0.104	0.060
319	Actinobacteriota;	100	0.29	ns-	ns-	565	5603	46	80	44	69	0.016	0.064	0.014	0.043
319	Enterorhabdus					565	5613	30	58	21	49	0.005	0.022	0.002	0.013
319						565	5616	49	59	47	55	0.009	0.012	0.008	0.008
330	Cyanobacteria;	92	0.19	–	ns-	581	5733	79	65	71	67	0.045	0.030	0.024	0.028
330	Gastranaerophilales					582	5751	79	77	74	76	0.088	0.084	0.055	0.067
330						582	5752	60	62	50	54	0.035	0.038	0.018	0.029

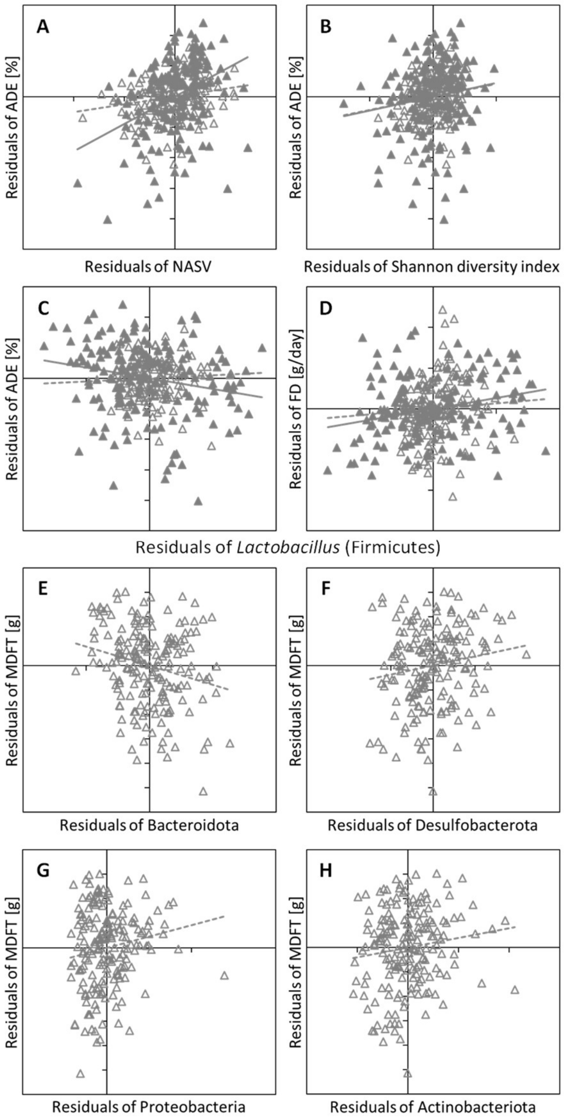

Fig. S5 Examples of the correlation between residual values of the feeding trial performance traits and microbial characteristics. A,B – between apparent digestive efficiency (ADE) and the number of amplicon sequence variants (N_{ASV}) and Shannon diversity index; **C,D** – between the ADE or effective digestion rate (FD) and the relative abundance of *Lactobacillus* (Firmicutes); **E-H** - between body mass change in the test with low-quality diet (MD_{FT}) measured in standard cages (i.e., the selection criterion in this experimental evolution model) and the relative abundances of four phyla.

References

Anderson M.J. 2017. Permutational Multivariate Analysis of Variance (PERMANOVA). Wiley StatsRef Stat Ref Online 1–15.

Benjamini Y. and Y. Hochberg. 1995. Controlling the false discovery rate - a practical and powerful approach to multiple testing. J R Stat Soc 57:289–300.

Bolyen E., J.R. Rideout, M.R. Dillon, N.A. Bokulich, C.C. Abnet, G.A. Al-Ghalith, H. Alexander, et al. 2019. Reproducible, interactive, scalable and extensible microbiome data science using QIIME 2. Nat Biotechnol 37:852–857.

Chakravarthy M. V. and F.W. Booth. 2004. Eating, exercise, and "thrifty" genotypes: Connecting the dots toward an evolutionary understanding of modern chronic diseases. J Appl Physiol 96:3–10.

Charles River. 2011. Helicobacter species technical sheet.

Christopherson M.R., J.A. Dawson, D.M. Stevenson, A.C. Cunningham, S. Bramhacharya, P.J. Weimer, C. Kendziorski, et al. 2014. Unique aspects of fiber degradation by the ruminal ethanologen Ruminococcus albus 7 revealed by physiological and transcriptomic analysis. BMC Genomics 15:1–13.

Glenn T.C., T.W. Pierson, N.J. Bayona-Vásquez, T.J. Kieran, S.L. Hoffberg, J.C. Thomas, D.E. Lefever, et al. 2019. Adapterama II: Universal amplicon sequencing on Illumina platforms (TaggiMatrix). PeerJ 2019.

Goller C.C. 2023. Quick-Start Protocol for DNeasy® PowerSoil® Pro Kit.

Hanhimäki E., P.C. Watts, E. Koskela, A.M. Hämäläinen, P. Koteja, T. Mappes, and A.M. Hämäläinen. 2022. Evolved high aerobic capacity has context-specific effects on gut microbiota. Front Ecol Evol 10:1–17.

Henderson N.D. 1997. Spurious associations in unreplicated selected lines. Behav Genet 27:145–154.

Hong Y., L. Sheng, J. Zhong, X. Tao, W. Zhu, J. Ma, J. Yan, et al. 2021. Desulfovibrio vulgaris, a potent acetic acid-producing bacterium, attenuates nonalcoholic fatty liver disease in mice. Gut Microbes 13:1–20.

Iwaszkiewicz-Eggebrecht E., P. Łukasik, M. Buczek, J. Deng, E.A. Hartop, H. Havnås, M. Prus-Frankowska, et al. 2023. FAVIS: Fast and versatile protocol for nondestructive metabarcoding of bulk insect samples. PLoS One 18:1–13.

Kohl K.D., J. Amaya, C.A. Passement, M.D. Dearing, and M.D. Mccue. 2014. Unique and shared responses of the gut microbiota to prolonged fasting: A comparative study across five classes of vertebrate hosts. FEMS Microbiol Ecol 90:883–894.

Kohl K.D., A. Brun, M. Magallanes, J. Brinkerhoff, A. Laspiur, J.C. Acosta, S.R. Bordenstein, et al. 2016. Physiological and microbial adjustments to diet quality permit facultative herbivory in an omnivorous lizard. J Exp Biol 219:1903–1912.

Lin H. and S. Das Peddada. 2020a. Analysis of compositions of microbiomes with bias correction. Nat Commun 11:1–11.

______. 2020b. Analysis of microbial compositions: a review of normalization and differential abundance analysis. npj Biofilms Microbiomes 6.

Lipowska M.M., E.T. Sadowska, U. Bauchinger, W. Goymann, B. Bober-Sowa, and P. Koteja. 2020. Does selection for behavioral and physiological performance traits alter glucocorticoid responsiveness in bank voles? J Exp Biol 223:1–14.

Lipowska M.M., E.T. Sadowska, K.D. Kohl, and P. Koteja. 2024. Experimental evolution of a mammalian holobiont? Genetic and maternal effects on the caecal microbiome in bank voles selectively bred for herbivorous capability. Ecol Evol Physiol 97:274–291.

Liu L., H. Wang, H. Zhang, X. Chen, Y. Zhang, J. Wu, L. Zhao, et al. 2022. Toward a deeper understanding of gut microbiome in depression: the promise of clinical applicability. Adv Sci 9:1–14.

Ma L., A. Eguchi, G. Liu, Y. Qu, X. Wan, R. Murayama, C. Mori, et al. 2024. A role of gut–brain axis on prophylactic actions of arketamine in male mice exposed to chronic restrain stress. Pharmacol Biochem Behav 238:173736.

Maiti U., E.T. Sadowska, K.M. Chrząścik, and P. Koteja. 2019. Experimental evolution of personality traits: open-field exploration in bank voles from a multidirectional selection experiment. Curr Zool

65:375–384.
Marizzoni M., T. Gurry, S. Provasi, G. Greub, N. Lopizzo, F. Ribaldi, C. Festari, et al. 2020. Comparison of Bioinformatics Pipelines and Operating Systems for the Analyses of 16S rRNA Gene Amplicon Sequences in Human Fecal Samples. Front Microbiol 11.
Marquina D., M. Buczek, F. Ronquist, and P. Lukasik. 2021. The effect of ethanol concentration on the morphological and molecular preservation of insects for biodiversity studies. PeerJ 9:1–22.
McNamara M.P., J.M. Singleton, M.D. Cadney, P.M. Ruegger, J. Borneman, and T. Garland. 2021. Early-life effects of juvenile Western diet and exercise on adult gut microbiome composition in mice. J Exp Biol 224.
Oksanen J., G.L. Simpson, F.G. Blanchet, R. Kindt, P. Legendre, P.R. Minchin, R.B. O'Hara, et al. 2022. Package "Vegan" Title Community Ecology Package. Cran 1–297.
Oren A. and G.M. Garrity. 2021. Valid publication of the names of forty-two phyla of prokaryotes. Int J Syst Evol Microbiol 71.
Palmas V., S. Pisanu, V. Madau, E. Casula, A. Deledda, R. Cusano, P. Uva, et al. 2021. Gut microbiota markers associated with obesity and overweight in Italian adults. Sci Rep 11:1–14.
Prodan A., V. Tremaroli, H. Brolin, A.H. Zwinderman, M. Nieuwdorp, and E. Levin. 2020. Comparing bioinformatic pipelines for microbial 16S rRNA amplicon sequencing. PLoS One 15:1–19.
Quast C., E. Pruesse, P. Yilmaz, J. Gerken, T. Schweer, P. Yarza, J. Peplies, et al. 2013. The SILVA ribosomal RNA gene database project: Improved data processing and web-based tools. Nucleic Acids Res 41:590–596.
Rivière A., M. Selak, D. Lantin, F. Leroy, and L. De Vuyst. 2016. Bifidobacteria and butyrate-producing colon bacteria: Importance and strategies for their stimulation in the human gut. Front Microbiol 7.
Sadowska E.T., K. Baliga-Klimczyk, K.M. Chrząścik, and P. Koteja. 2008. Laboratory model of adaptive radiation: a selection experiment in the bank vole. Physiol Biochem Zool 81:627–640.
Sadowska E.T., C. Stawski, A. Rudolf, G. Dheyongera, K. Baliga-Klimczyk, P. Koteja, and K.M. Chrząścik. 2015. Evolution of basal metabolic rate in bank voles from a multidirectional selection experiment. Proc R Soc B 282:20150025.
SAS Institute Inc. 2011. Base SAS® 9.3 Procedures Guide. Cary, NC: SAS Institute Inc.
Schwarzer M., K. Makki, G. Storelli, I. Machuca-Gayet, D. Srutkova, P. Hermanova, M.E. Martino, et al. 2016. Lactobacillus plantarum strain maintains growth of infant mice during chronic undernutrition. Science (80-) 351:854–857.
Shinohara A., E. Uchida, H. Shichijo, S.H. Sakamoto, T. Morita, and C. Koshimoto. 2016. Microbial diversity in forestomach and caecum contents of the greater long-tailed hamster Tscherskia triton (Rodentia: Cricetidae). Mamm Biol 81:46–52.
Singh S.B., A. Carroll-Portillo, and H.C. Lin. 2023. Desulfovibrio in the Gut: The Enemy within? Microorganisms 11:1–21.
Zhang J., K. Kobert, T. Flouri, and A. Stamatakis. 2014. PEAR: A fast and accurate Illumina Paired-End reAd mergeR. Bioinformatics 30:614–620.
