# Supplementary material for: Experimental evolution of a mammalian holobiont: bank voles selected for herbivorous capability evolved distinct and robust gut bacterial communities
Source: ISME Commun. 2025 Sep 11;5(1):ycaf160. doi: 10.1093/ismeco/ycaf160 (PMC12516954; doi:10.1093/ismeco/ycaf160)
Supplement: Supporting_information_ycaf160 [file supporting_information_ycaf160.docx]

**Supporting information**

**1) Lipowska_ Suppl_MethResFigsTablesR1-2.pdf**: pdf file containing Supplementary Methods: details of the animal model and methods (with Fig. S1); Supplementary Results: results concerning the microbiome including [Clostridium] innocuum (with Fig. S2); effect of body mass on the host traits (Fig. S3-4); abundances of particular bacterial taxa and ASVs, and their correlations with host traits (with Tables R1, R2, Fig. S5).

**2) Lipowska_ Suppl_TablesS1-S16.xlsx:** xlsx file containing 16 sheets with supplementary Tables S1 – S16 and a Legend sheet, presenting information about the number of voles at subsequent stages of the experiment (S1), information on ASVs and taxonomy (S2-3), descriptive statistics of physiological traits and alpha diversity metrics (S4), results of ANCOVA models for physiological traits and alpha diversity metrics (S5) and PERMANOVA on UniFrac distances (S6), descriptive statistics of prevalence and abundance of the bacterial taxa and ASVs (S7-8), comparisons of relative and absolute abundances of bacterial taxa and ASVs (S9-14), and analyses of phenotypic correlations between the bacterial characteristics and physiological traits (S15-16).
